# Semi‐Crystalline Ruthenium Catalyst for Zero‐Drag Hydrogen Production from Hybrid Alkaline Seawater Electrolysis

**DOI:** 10.1002/advs.202507848

**Published:** 2025-06-27

**Authors:** Dongquan Yang, Rui Yang, Huayi Zeng, Jiajun Luo, Shuangjuan Shen, Yiyin Huang, Yuanyuan Sun

**Affiliations:** ^1^ College of Physics and Energy Fujian Normal University Fujian Provincial Key Laboratory of Quantum Manipulation and New Energy Materials Fuzhou 350117 P. R. China; ^2^ Fujian Provincial Engineering Technology Research Center of Solar Energy Conversion and Energy Storage Fuzhou 350117 P. R. China; ^3^ School of Materials and Chemistry Anhui Agricultural University Hefei 230036 P. R. China; ^4^ Institute of Innovation Materials and Energy College of Chemistry & Chemical Engineering Yangzhou University Yangzhou 225002 P. R. China

**Keywords:** bifunctional, hydrogen evolution, interface, ruthenium, seawater electrolysis

## Abstract

Hydrazine‐assisted hybrid alkaline seawater electrolysis offers a dual‐functional platform for environmentally benign remediation of toxic hydrazine and energy‐autonomous hydrogen generation. Addressing the critical need for simplified system integration, a single‐metal bifunctional catalyst is developed by modulating electronic metal‐support interactions (EMSI) to construct semi‐crystalline Ru domains with metastable crystalline‐amorphous interfaces. The optimized catalyst achieves ultralow overpotentials of 21.5 mV (hydrogen evolution) and 254 mV (hydrazine oxidation) at 10 mA cm⁻^2^, alongside spontaneous hydrazine decomposition at open‐circuit potential. This synergy enables near‐zero energy input for electrolysis, evidenced by a steep polarization slope (1.235 A cm⁻^2^ V⁻¹), which surpasses conventional hybrid systems. Density functional theory (DFT) calculations reveal that amorphous Ru sites near the interface induce charge redistribution, which partially optimizes the free energy changes associated with adsorption ^*^H and the dehydrogenation process from ^*^N₂H₄ to ^*^N₂H₃. This is accompanied by a transformation of the rate‐determining step into the ^*^N₂H → ^*^N₂ pathway, thereby advancing the kinetics of the bifunctional hydrogen evolution reaction/hydrazine oxidation reaction (HER/HzOR) reactions. The work redefines catalyst design paradigms by leveraging interfacial metastability, bridging pollutant elimination with high‐efficiency hydrogen economies.

## Graphical abstract

Tune metal‐support interaction to induce formation of crystalline/amorphous Ru interface towards zero‐drag hydrogen generation from hybrid alkaline seawater electrolysis.

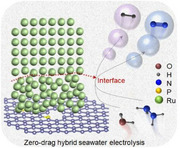

## 1 Introduction

The goal of implementing carbon neutrality has stimulated the development of diversified new energy technologies, among which electrochemical water splitting is recognized as a promising technology for production of hydrogen, not only a clean energy source with high gravimetric energy density (142 MJ kg^−1^),^[^ ^1^ ^]^ but also an important chemical feed for industrial production. Electrochemical water splitting in alkaline media was widely applied, which includes hydrogen evolution reaction (HER) and oxygen evolution reaction (OER), while chlorine evolution reaction (CIER) would further be involved when using earth‐abundant seawater as the electrolyte to reduce cost of practical applications.^[^ ^2^ ^]^ However, the sluggish 4e^−^‐transfer process for OER,^[^ ^3^ ^]^ and the deleterious effects derived from Cl^−^‐ion chemistry during seawater electrolysis^[^ ^4^, ^5^ ^]^ have propelled researchers to exploit thermodynamically more favorable anode reactions to couple HER for energy‐saving H_2_ production. Hitherto electrooxidation of alcohols,^[^ ^6^ ^]^ urea,^[^ ^7^ ^]^ amines,^[^ ^8^ ^]^ tetrahydroisoquinoline,^[^ ^9^ ^]^ iodide,^[^ ^10^ ^]^ glucose,^[^ ^11^ ^]^ chitin,^[^ ^12^ ^]^ sulfur,^[^ ^13^ ^]^ furfural,^[^ ^13^ ^]^ and hydrazine,^[^ ^14^ ^]^ has been reported. Thereinto, hydrazine (N_2_H_4_), a toxic and corrosive pollutant in industrial wastewater, could serves as anode reactant to produce harmless nitrogen and water via hydrazine oxidation reaction (HzOR, 4OH^−^ + N_2_H_4_→4e^−^ + 4H_2_O + N_2_) at a lower equilibrium potential (−0.33 V vs reversible hydrogen electrode (RHE)).^[^ ^14^ ^]^ Employing hydrazine in alkaline hybrid seawater electrolysis is not only beneficial for addressing the aforesaid issues, but also helps to maintain environmental sustainability.^[^ ^2^ ^]^ Developing highly efficient bifunctional electrocatalysts of single metal for HER and HzOR is crucial for simplifying system design and maintenance of this hybrid seawater electrolysis technology, yet remains a challenge.

The challenge primarily roots in the sluggish kinetics for both reactions: i) the extra water dissociation process for subsequent Volmer step (H_2_O + e^−^ + ^*^→ H^*^ + OH^−^) in alkaline HER is needed but more sluggish in kinetics compared to acidic media,^[^ ^3^ ^]^ adding additional resistance for HER; ii) the stepwise dehydrogenation processes, such as demanding conversion from N_2_H_3_^*^ to N_2_H_2_^*^,^[^ ^2^ ^]^ and the steric hindrance effect of N_2_H_4_ molecule,^[^ ^15^ ^]^ cause a significant hurdle in the proceeding of HzOR. A great deal of non‐precious metal nanoparticles and compounds, such as phosphides and nitrides,^[^ ^16^, ^17^ ^]^ have been developed, most of which show HER activity inferior to Pt, and they may cause cleavage of N─N bond^[^ ^18^ ^]^ to form the undesired ammonia via incomplete oxidation of HzOR.^[^ ^14^ ^]^ Platinum (Pt) normally serves as the benchmark material for HER,^[^ ^19^ ^]^ however, it underperforms in HzOR, and the high cost limits its practical applications. Ruthenium (Ru) with its 4% price of Pt,^[^ ^20^ ^]^ has a similar hydrogen adsorption energy to Pt and been further explored to promote HzOR via accelerating dehydrogenation processes.^[^ ^2^, ^21^ ^]^ Although Ru has gained ever‐increasing interest for its potential to achieve zero‐drag HER/HzOR applications based on the potential coincidence region between HER and HzOR,^[^ ^22^ ^]^ this goal has been not explicitly implemented especially confronting hybrid seawater electrolysis.

Interface engineering is a potent approach to achieving novel properties that enhance electrocatalytic processes, owing to the diverse interfacial chemistry involved. This includes improved charge transfer, modified interfacial electronic states, potential built‐in electric fields, dislocations, and stress at the interfaces.^[^ ^1^, ^23^, ^24^, ^25^ ^]^ In the case of a simple supported Ru catalyst, the primary type of interface to be engineered with enhanced HER/HzOR efficiency lies at Ru moieties with distinct structures. The electronic metal‐support interaction (EMSI) was usually used to facilitate uniform dispersion of metal particles and alter the electron transfer characteristics.^[^ ^26^, ^27^, ^28^ ^]^ In spite of the fact that it provides an attraction for mediating metal deposition sites (on metal or substrate surface), leading to the possible formation of different metal structures and interfaces. However, there is still less attention in this area as far as we know. Carbon materials have been widely applied for supporting metal material via EMSI, while EMSI of pure carbon is relatively weak for loading metal catalyst,^[^ ^29^, ^30^ ^]^ probably causing metal particle aggregation, detachment, and even dissolution^[^ ^15^ ^]^ and suggesting modification of carbon support is requisite.

Here, we demonstrate a tuning EMSI strategy by using sodium hypophosphite‐treated carbon, showing enhanced EMSI compared to pure carbon to induce the formation of semi‐crystalline Ru. The catalyst with abundant amorphous/crystalline Ru interfaces was disclosed to promote bifunctional HER/HzOR performance effectively. It achieves the ability of spontaneously decomposing hydrazine and zero‐drag processes for hybrid seawater electrolysis, witnessed by the LSV region from HER to HzOR processes with the high slope of 1.235 A cm^−2^ V^−1^ in alkaline seawater electrolyte. It also shows a robust catalytic durability at various currents and temperatures, surpassing the benchmark Pt/C catalyst (20 wt.%). Physicochemical and theoretical analysis discloses that amorphous Ru sites near the interface can optimize the electronic structure to make a more thermoneutral H adsorption/desorption for HER and reduce the free energy change for the dehydrogenation process from ^*^N_2_H_4_ to ^*^N_2_H_3_, accompanied with transformation of the rate‐determining step into the ^*^N_2_H → ^*^N_2_ pathway, thereby advancing the kinetics of HER/HzOR bifunctionality and enabling hybrid seawater electrolysis for H_2_ production requiring only a voltage of 25 mV to reach 10 mA cm^−2^.

## 2 Results and Discussion

**Figure 1:**
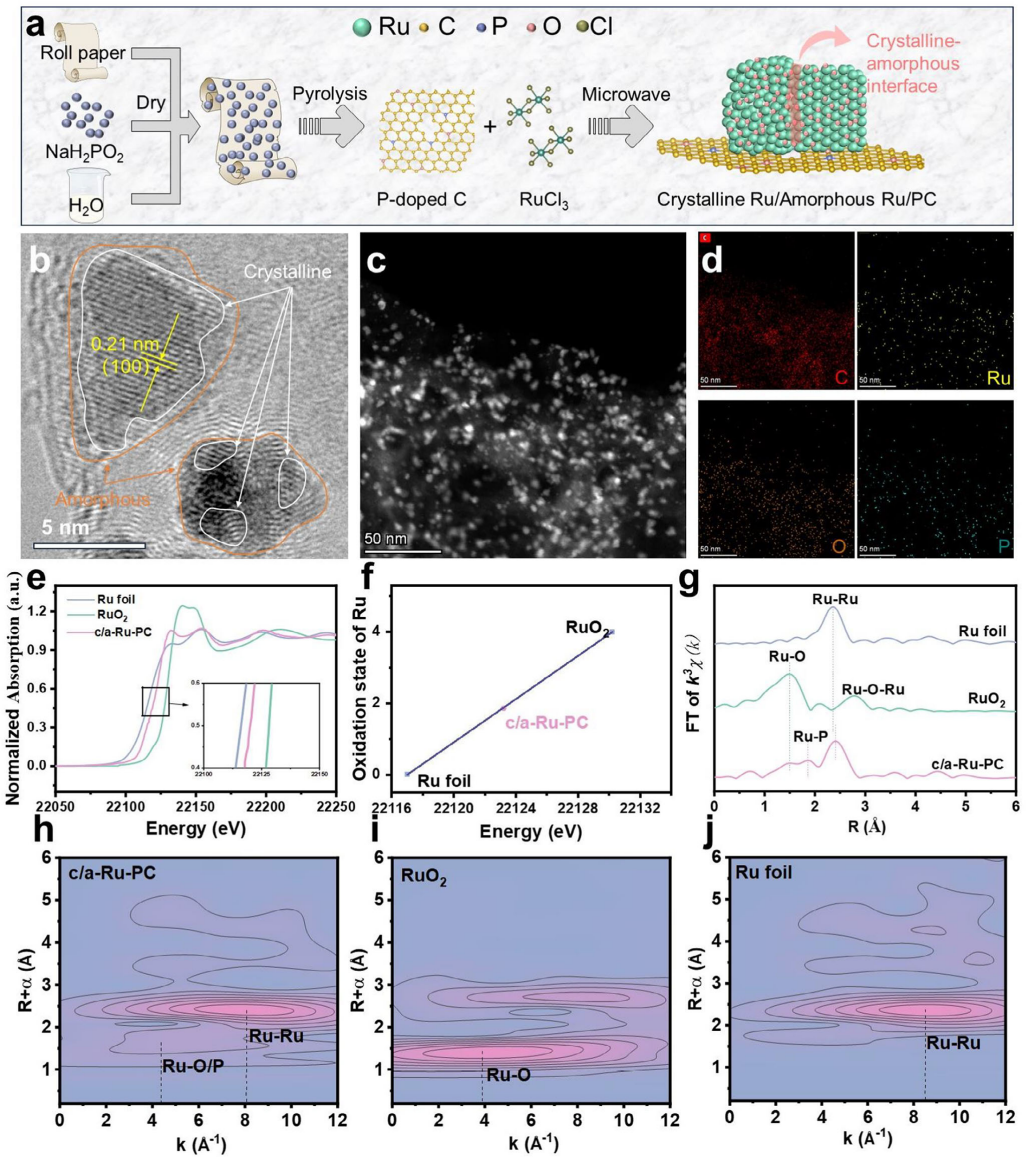
Synthesis and characterizations on nano and atomic structure. (a) Schematic illustration for preparing c/a‐Ru/PC catalyst. (b) TEM and (c) high‐angle annular dark field image of c/a‐Ru/PC sample. (d) Elemental mapping images for C, Ru, P, and O. (e) Ru K‐edge X‐ray absorption near‐edge structure (XANES) spectra and (g) their Fourier transforms (FT) of c/a‐Ru/PC, Ru foil, and RuO_2_ samples. (f) Chemical valence of Ru atom in c/a‐Ru/PC. Wavelet transform (WT) of k^3^‐weighted extended X‐ray absorption fine structure (EXAFS) signals of Ru K‐edge for (h) c/a‐Ru/PC, (i) RuO_2_, and (j) Ru foil materials. a.u., arbitrary units; k, wave vector.

**Figure** **1**a illustrates the synthesis pathway for the crystalline‐amorphous Ru/phophorus‐doped carbon (c/a‐Ru/PC) catalyst. Transmission electron microscopy (TEM) analysis (Figure 1b; Figure S1a,b, Supporting Information), combined with selected area electron diffraction (SAED) measurement (Figure S1c, Supporting Information), confirms the formation of highly dispersed crystalline‐amorphous Ru nanoparticles (4–8 nm), contrasting with aggregated Ru particles on unmodified carbon (Figure S2, Supporting Information). This structural control originates from sodium hypophosphite‐induced carbon etching and phosphorus doping (cf. ammonia‐carbon reactions^[^ ^31^ ^]^), creating a defective, porous architecture (Figure S1, Supporting Information) that enhances electronic interaction between metal and support during synthesis.^[^ ^32^ ^]^ Similar phenomenon was observed between Ru species and amorphous VO_x_ support.^[^ ^33^ ^]^ High‐resolution TEM reveals characteristic 0.21 nm lattice fringes corresponding to metallic Ru (100) planes (Figure 1b). High angle ring dark field‐scanning transmission electron microscopy‌ (HAADF‐STEM) and energy dispersive spectroscopy (EDS) mapping (Figure 1c,d) demonstrate homogeneous dispersion of Ru, P, and O across the carbon matrix. Inductively coupled plasma atomic emission spectrometer (ICP‐AES) quantification confirms comparable Ru loading in c/a‐Ru/PC (5.16 wt.%) and Ru/C (4.80 wt.%).

Ru K‐edge X‐ray absorption fine structure (XAFS) analysis elucidates the interfacial interaction and bonding types of c/a‐Ru/PC. The X‐ray absorption near‐edge structure (XANES) spectrum of c/a‐Ru/PC aligns closely with Ru foil (Figure 1e), and its absorption edge energy lies between RuO_2_ and Ru foil, with a marginally elevated white‐line intensity (Figure 1e), indicative of partial oxidation (average oxidation state ≈ +1.9; **Figure** **3**f). This mixed valence state arises from coordination of Ru with oxygen and phosphorus, attributed to heteroatom doping‐induced electronic modulation. Such bonding likely stabilizes metastable Ru sites while preserving metallic domains critical for catalytic functionality. Extended X‐ray absorption fine structure (EXAFS) analysis reveals the complex coordination environment of Ru in c/a‐Ru/PC (Figure 1g; Figures S3–S8, Supporting Information). Compared to Ru foil (Ru─Ru: 2.36 Å) and RuO_2_ (Ru─O: 1.49 Å; Ru─O─Ru: 2.78 Å),^[^ ^20^, ^34^ ^]^ c/a‐Ru/PC exhibits hybridized scattering paths: elongated Ru─Ru bonds (2.42 Å) attributed to oxygen‐induced lattice distortion and amorphous‐phase disordering, alongside Ru─O (1.49 Å) and a distinct Ru─P interaction at 1.85 Å.^[^ ^21^ ^]^ The latter's shortened bond length relative to crystalline RuP suggests strong interfacial Ru─P covalent bonding, which stabilizes amorphous Ru domains via metal‐support electronic coupling. Quantitative fitting (Table S1, Supporting Information) confirms a mixed coordination environment (Ru‐Ru: 4.9; Ru─P: 1.2; Ru─O: 3.3), further validated by wavelet transform (WT) analysis of k^3^‐weighted EXAFS spectra (Figure 1h–j). This diversified bonding rationalizes the structural metastability and catalytic versatility of the c/a‐Ru/PC system.

**Figure 2:**
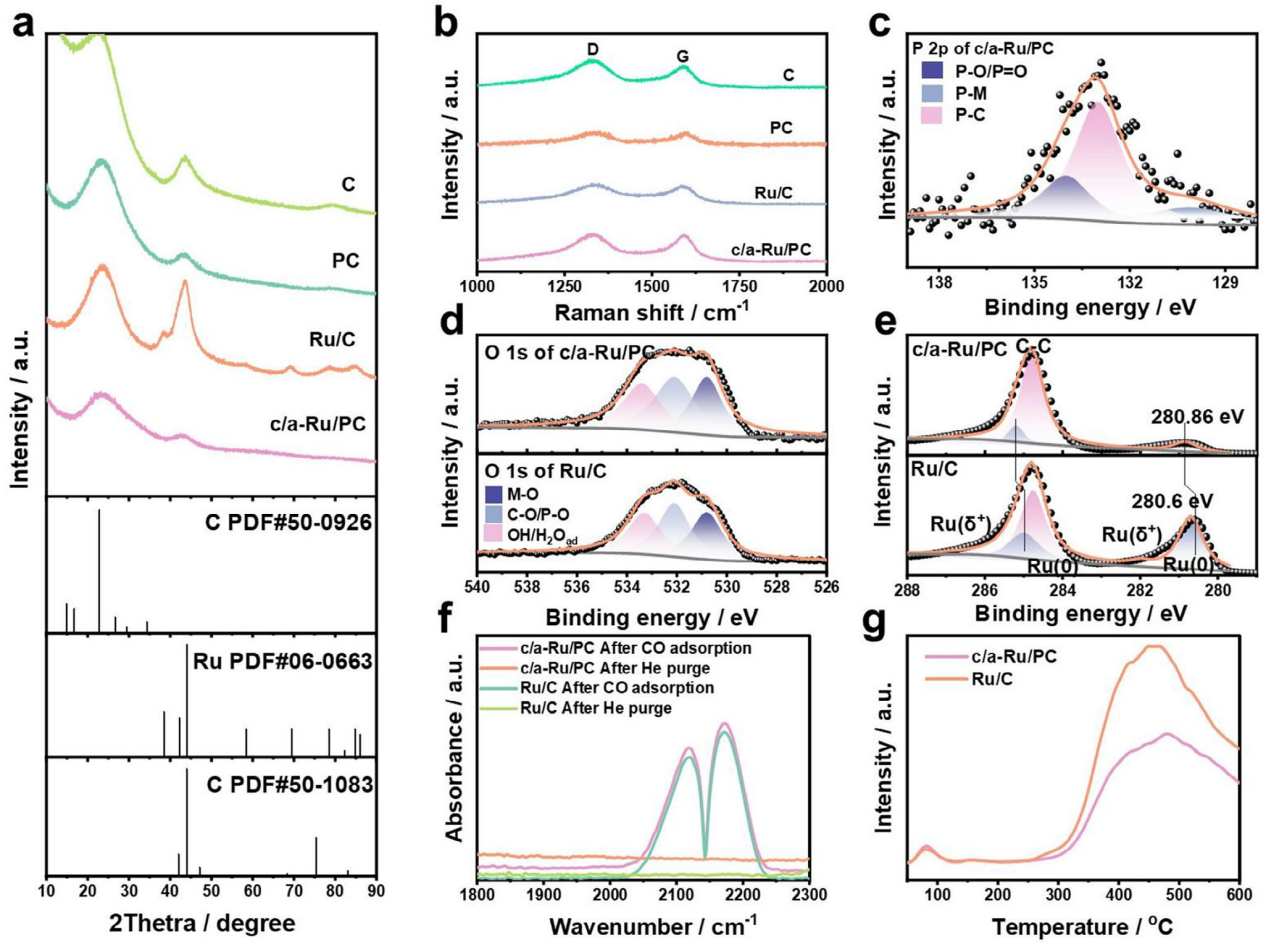
Structural characterization of catalysts. (a) XRD patterns and (b) Raman spectra of different materials. Deconvolution in the high‐resolution X‐ray photoelectron spectroscopy (XPS) spectra of (c) P 2p region for c/a‐Ru/PC, (d) O 1s region, and (e) Ru 3d and C 1s regions for c/a‐Ru/PC and Ru/C samples. (f) In situ diffuse reflection infrared Fourier‐transform spectroscopy (DRIFT) spectra acquired after CO adsorption and evacuation with helium on c/a‐Ru/PC and Ru/C samples. (g) Temperature‐programmed H_2_ desorption (H_2_‐TPD) of Ru/PC and Ru/C samples.

X‐ray diffraction (XRD) analysis elucidates the EMSI and crystalline evolution of Ru (**Figure** **2**a; Figure S9, Supporting Information). The (120) and (012) carbon planes (JCPDS 50–0926/1083) exhibit attenuated (012) peak intensity in PC and c/a‐Ru/PC versus pristine carbon and Ru/C, confirming sodium hypophosphite‐induced structural degradation during pyrolysis. Elevated hypophosphite concentrations and temperatures further diminish this peak via analogous etching mechanisms. While Ru/C displays distinct Ru(100)/(102)/(110)/(103)/(112) diffraction peaks (JCPDS 06–0663), c/a‐Ru/PC under mild etching retains only a weakened Ru(100) signal, which diminishes with intensified (012) plane destruction. These trends correlate with harsher etching protocols amplifying EMSI‐driven Ru disorder, underscoring the interplay between support modification and metallic phase amorphization.

Raman spectroscopy (Figure 2b) reveals distinct D (1330 cm⁻¹) and G (1590 cm⁻¹) bands across all catalysts, corresponding to defect‐associated A₁g graphite vibrations and defect‐free sp^2^ carbon E_2_g modes,^[^ ^35^ ^]^ respectively. The I_D_/I_G_ ratios (C:1.05; PC:1.08; Ru/C:1.02; c/a‐Ru/PC:1.07) demonstrate enhanced defect density in PC and c/a‐Ru/PC relative to pristine carbon and Ru/C. This elevated disorder stems from sodium hypophosphite‐induced carbon etching during synthesis, which disrupts the homogeneity of the carbon matrix, generating more defect sites and amplifying EMSI. The correlation between defect generation and hypophosphite treatment underscores EMSI's critical role in modulating carbon substrate adsorption capacity and interfacial structure.

X‐ray photoelectron spectroscopy (XPS) survey spectra confirm Ru, O, C, and P in the c/a‐Ru/PC sample (Figure S10a, Supporting Information). Deconvolution of the P 1s spectrum for c/a‐Ru/PC (Figure 2c) reveals three distinct P species: P─M (130.1 eV), P─C (133.0 eV), and P─O/P═O (134.0 eV),^[^ ^36^ ^]^ corroborating EXAFS‐derived interfacial Ru─P bonding and phosphorus doping. Phosphorus atom doping could modify the electronic structure of the carbon matrix and induce charge redistribution, critical for Ru dispersion. O 1s analysis (Figure 2d; Figure S10c, Supporting Information) identifies coexisting M‐O, C‐O, and OH/H_2_O_ad_ species,^[^ ^37^ ^]^ with Ru‐O contributions at 32.5% (Ru/C) and 31.8% (c/a‐Ru/PC). Despite lower total O content in c/a‐Ru/PC (6.61% vs 9.18% for Ru/C), its less Ru─O fraction highlights efficient reduction of Ru under stronger EMSI during synthesis. Ru 3d/C 1s spectra (Figure 2e; Figure S10d, Supporting Information) confirm dominant metallic Ru(0) with minor Ru(δ+), while a 0.26 eV positive Ru peak shift in c/a‐Ru/PC aligns with XANES‐derived oxidation states, conclusively evidencing EMSI‐driven electronic modulation.

In situ diffuse reflection infrared Fourier‐transform spectroscopy (DRIFTs) analysis (Figure 2f) reveals attenuated interfacial coupling between Ru and PC, evidenced by transient CO adsorption peaks (2220–2250 cm⁻¹) absent in He‐purged conditions, in contrast to the Ru‐TiN system with strong CO‐Ru^n+^ interactions (≈1917 cm⁻¹).^[^ ^38^ ^]^ The minimal Ru 3d XPS shift here conjointly confirms weaker EMSI in Ru/PC versus Ru‐TiN, while it is stronger compared to Ru/C. The PC substrate could effectively anchor metal atoms while promoting disordered atomic deposition and stabilization as revealed above. Temperature‐programmed H_2_ desorption (H_2_‐TPD) profiles (Figure 2g) further demonstrate suppressed H₂ adsorption at Ru sites (<160 °C) and interfaces (>300 °C) for c/a‐Ru/PC.^[^ ^38^ ^]^ This diminished high‐temperature desorption peak, despite enhanced interfacial density, reflects optimized H_2_ desorption kinetics—a critical factor for HER efficiency. This relation between low H₂ adsorption ability of c/a‐Ru/PC and highly‐dispersed Ru with semi‐crystalline structure underscores its superior interfacial design for facilitating H_2_ release.

**Figure 3:**
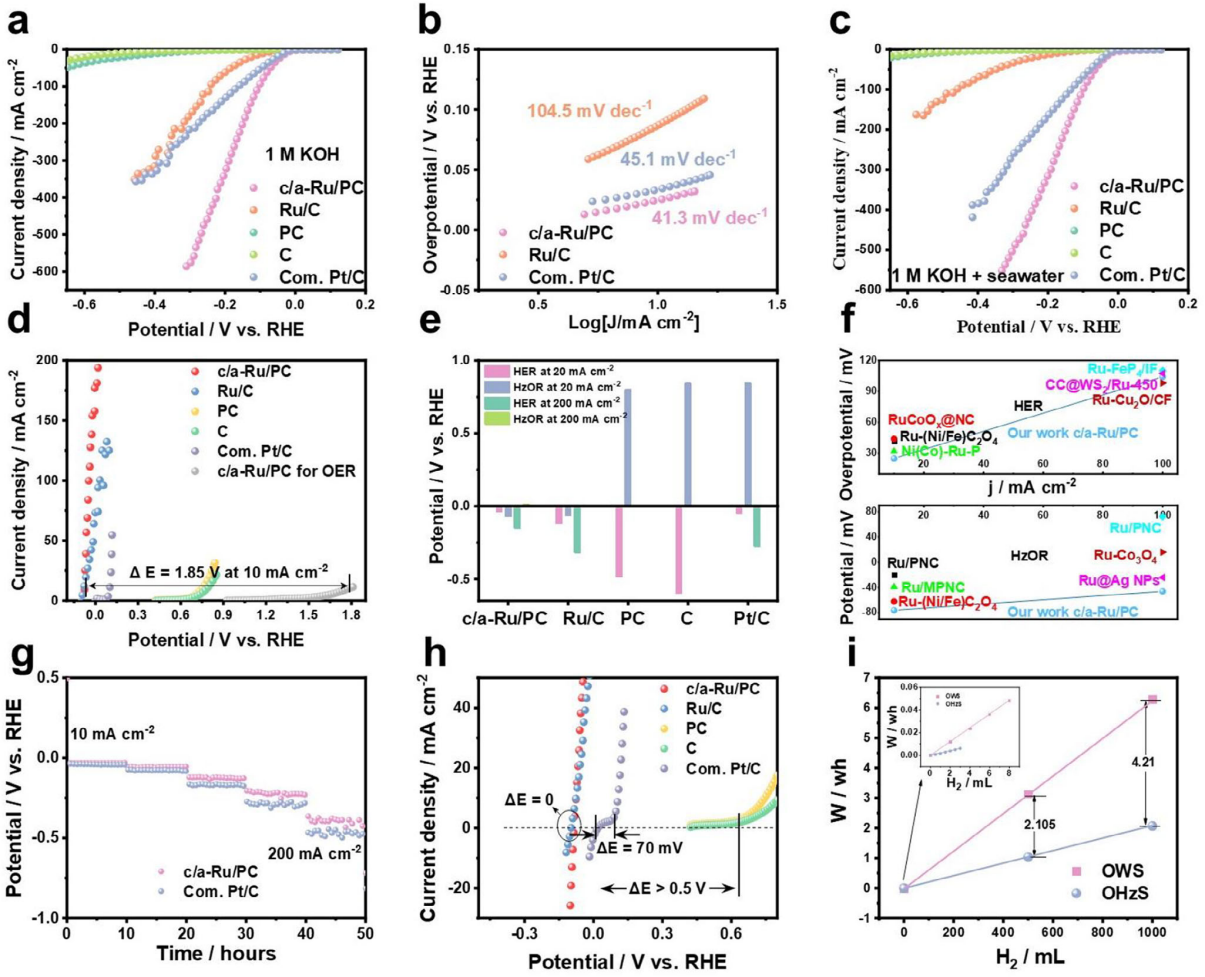
Electrocatalytic HER/HzOR performance of catalysts. (a) LSV curves (85% iR‐correction) of the as‐prepared catalysts and commercial Pt/C in 1 m KOH solution. (b) Tafel plots of different catalysts. (c) LSV curves of different catalysts in 1 m KOH + seawater solution. (d) Anode oxidation LSV curves for HzOR and OER on different catalysts. (e) Comparison in potentials versus RHE on HER and HzOR on the catalysts. (f) Comparison in bifunctional performance of c/a‐Ru/PC with other catalysts reported.^[^ ^4^, ^13^, ^20^, ^21^, ^23^, ^39^, ^40^, ^41^ ^]^ (g) Chronopotentiometry at varied currents in 1 m KOH+seawater electrolyte. (h) LSV curves for different catalysts in 1 m KOH + 1 m N_2_H_4_ + seawater solution, in which c/a‐Ru/PC and Ru/C have zero‐drag hybrid seawater electrolysis from HER to HzOR regions. (i) Estimation the power consumption when generating 0.5 and 1.0 L of H_2_ by fitting W versus the amount of H_2_ curve.

Electrochemical HER performance was evaluated in KOH electrolyte using RHE‐calibrated potentials (Figure S11, Supporting Information). Linear scan voltammogram (LSV) polarization analysis guided synthesis optimization, identifying the optimal c/a‐Ru/PC catalyst (0.58 at.% P; 1000 °C pyrolysis, 100 s microwave Ru deposition; Figures S12–S13, Supporting Information). c/a‐Ru/PC exhibits exceptional HER activity with ultralow overpotentials of 24.7 mV (10 mA cm⁻^2^), 104.8 mV (100 mA cm⁻^2^), and 226.7 mV (400 mA cm⁻^2^), surpassing Pt/C (33.7/161.9/360.9 mV) and Ru/C (Figure 3a). A minimal Tafel slope (41.3 mV dec⁻¹ vs Pt/C:45.1; Ru/C:104.5; Figure 3b) and near‐unity Faradaic efficiency (99% at 100 mA cm⁻^2^; Figure S14, Supporting Information) position it among top‐tier HER catalysts (Table S2, Supporting Information). Activity stems from Ru active sites amplified by synergistic crystalline‐amorphous interface engineering. Crucially, seawater electrolysis (Pingtan source; Figure S15, Supporting Information) reveals negligible ion interference (F⁻, SO₄^2^⁻, NO₃⁻, Mg^2^⁺, Ca^2^⁺),^[^ ^42^ ^]^ with c/a‐Ru/PC maintaining superior performance (21.5/99.6/244.3 mV at 10/100/400 mA cm⁻^2^; Figure 3c), underscoring its robustness in complex electrolytes.

The c/a‐Ru/PC catalyst demonstrates exceptional HzOR activity, achieving a potential of −0.076 V at 10 mA cm⁻^2^ in hydrazine‐containing electrolyte—outperforming Pt/C (0.110 V) and rivaling advanced benchmarks (Figure 3d; Table S3, Supporting Information). Substituting OER (1.77 V @ 10 mA cm⁻^2^) with HzOR induces a 1.85 V anode potential reduction, a trend mirrored across tested catalysts (Ru/C, PC, C). Spontaneous hydrazine splitting (Video S1, Supporting Information) and low coupled HER/HzOR overpotentials (−40.4 mV @ 20 mA cm⁻^2^; −150.2 mV @ 200 mA cm⁻^2^ for HER; −70.3/16.1 mV for HzOR; Figure 3e) underscore its energy‐efficient hybrid electrolysis capability. Copper underpotential deposition (Cu‐UPD) analysis (Figure S16, Supporting Information) reveals superior active site density (1.88 × 10⁻^3^ vs Pt/C:1.195 × 10⁻^3^ mol g⁻¹) and HER/HzOR turnover frequencies (TOF: 4.71/14.9 s⁻¹ vs Pt/C:1.29/0.042 s⁻¹) in KOH solution without and with N_2_H_4_ at −0.05 and 0 V, respectively, attributed to enhanced noble metal dispersion and interfacial synergy. These metrics position c/a‐Ru/PC as a top‐tier bifunctional catalyst, benchmarking against state‐of‐the‐art systems (Figure 3f).

Chronoamperometry reveals superior electrocatalytic robustness of c/a‐Ru/PC versus Pt/C. At HER‐relevant currents, c/a‐Ru/PC maintains potentials closer to 0 V (Figure 3g), with performance gaps widening at higher current densities. Besides, c/a‐Ru/PC maintains high stability at different electrolyte temperatures, and the 150 h test (Figure S17, Supporting Information). Similarly, in HzOR durability tests (Figure S18, Supporting Information), c/a‐Ru/PC exhibits lower potentials than Pt/C at 10–50 mA cm⁻^2^, in combination with the 120 h test, confirming exceptional operational stability. Post‐stability characterization (Figures S19, S20, Supporting Information) identifies phosphorus depletion and carbon support hydrogenation (C_x_H_γ_ formation) as contributors to minor activity decay. Concurrent ICP‐OES analysis of the electrolyte revealed Ru concentrations of 0.0014 mg L^−1^ after 150 h HER testing and 0.0022 mg L^−1^ after 120 h HzOR testing, confirming minimal dissolution (only approximately 1.1% and 1.7%). c/a‐Ru/PC's retained performance—attributed to its semi‐crystalline Ru architecture—outperforms Pt/C, underscoring the critical role of metastable Ru domains in preserving bifunctional (HER/HzOR) activity under prolonged operation.

Replacing the OER with the thermodynamically favorable HzOR (*E*  = −0.33 V vs RHE) enables near‐zero‐resistance electrolysis when paired with efficient bifunctional catalysts. In alkaline freshwater and seawater electrolytes, c/a‐Ru/PC achieves seamless integration of HER and HzOR, exhibiting a negligible onset potential difference (0 V) and linear HER‐HzOR transition (Figure S21, Supporting Information; Figure 3h). This contrasts sharply with Pt/C (82/70 mV onset gap) and carbon‐based catalysts (>0.7 V gap). The c/a‐Ru/PC demonstrates superior bifunctional kinetics, evidenced by steeper polarization slopes (3.306 A cm⁻^2^ V⁻¹ in freshwater; 1.235 A cm⁻^2^ V⁻¹ in seawater) versus Ru/C (0.497/0.430 A cm⁻^2^ V⁻¹). Such kinetic acceleration translates to significant energy savings: 2.105–4.21 Wh conserved per 0.5–1.0 L H_2_ produced via hydrazine‐assisted electrolysis versus conventional seawater splitting (Figure 3i). These metrics underscore the transformative potential of c/a‐Ru/PC in enabling energy‐autonomous hydrogen generation through synergistic HER/HzOR catalysis.

Mechanistic analyses elucidate the structural and interfacial origins of c/a‐Ru/PC's superior electrocatalytic performance. Open‐circuit potential (OCP) measurements (Figure S22, Supporting Information) reveal a higher steady‐state potential (1.02 V vs RHE) for c/a‐Ru/PC than Ru/C (0.98 V), indicative of enhanced water dissociation capacity—a critical step for alkaline HER.^[^ ^43^ ^]^ Electrochemical impedance spectroscopy (Figure S23 and Tables S4, S5, Supporting Information) demonstrates accelerated charge transfer kinetics, with lower charge transfer resistance (*R*_ct_: 6.63 Ω for HER; 25.5 Ω for HzOR) and Warburg impedance (*R*₁: 6.76/1.48 Ω) versus Ru/C (75.2/29.5 Ω; 9.17/6.1 Ω), confirming efficient electron/mass transport. Comparable electrochemical active areas (C_dl_: ≈35–37 mF cm⁻^2^; Figures S24, S25, Supporting Information) between c/a‐Ru/PC and PC, despite higher Ru dispersion in the former, highlight phosphorus‐induced porosity and defect engineering as key to exposing active sites. Thiocyanate poisoning (Figures S26, S27, Supporting Information) validates Ru as the primary active center, with SCN⁻ drastically suppressing HER/HzOR activity.^[^ ^44^ ^]^ Superhydrophilicity (Figure S28, Supporting Information), evidenced by instantaneous water droplet absorption, synergizes with OCP‐derived water dissociation kinetics, completing a self‐consistent mechanistic framework linking site‐water interaction to catalytic excellence.

Density functional theory (DFT) calculations were systematically employed to elucidate the spatial distribution of active sites and their thermodynamic influence on both HER and HzOR. Building upon the structural evidence from XRD and TEM analyses, we constructed three distinct heterointerface models: i) a crystalline Ru(100)‐phosphorus‐doped carbon hybrid configuration, ii) a crystalline Ru(100)‐amorphous Ru hybrid configuration, and iii) an amorphous Ru‐PC substrate composite system, as illustrated in Figure S29 (Supporting Information). The crystalline Ru(100) facet served as the reference model for comparative analysis.

**Figure 4:**
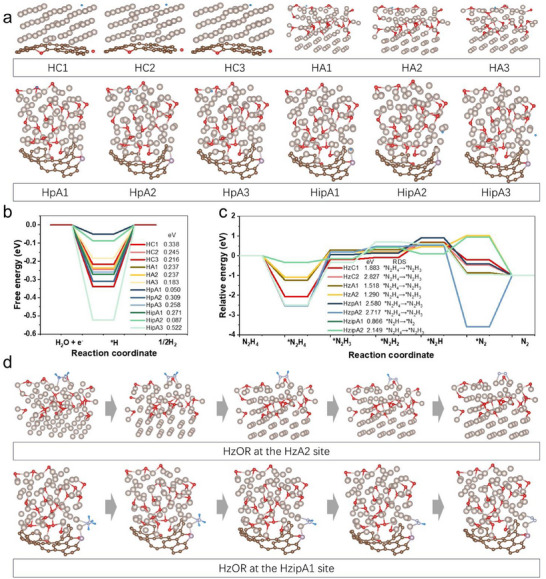
DFT calculations of HER/HzOR activities for the optimized structure sites on different Ru sites. (a) A schematic of H adsorption on crystalline and disorder Ru active sites for HER. The calculated free energy profiles of (b) H adsorption for HER, (c) adsorption of N_x_H_y_ species for HzOR. The insets in (b) and (c) show Free energy change of the rate control step. d) N_x_H_y_ intermediate adsorption on HzA2 and HzipA1 models for HzOR, respectively. Ru, C, O, N, P, and H elements are marked by gray, brown, red, cambridge blue, purple, and blue respectively. The names of HC1, HA2, HzpA1, and HzipA2 represent the first kind crystalline site for HER, the second kind amorphous site for HER, the first kind amorphous Ru site on Ru‐PC for HzOR, and the second kind interface amorphous Ru site at Ru‐PC for HzOR, respectively. Others were named in a similar fashion.

For HER activity assessment, hydrogen adsorption free energy (*ΔG* *_H_*) was identified as the critical descriptor, where optimal catalytic performance occurs near the thermodynamic neutral point *(ΔG_H_* ≈ 0). Our systematic sampling across 12 representative sites (3 crystalline Ru sites, 9 amorphous sites, **Figure** **4**a,b) revealed distinct electronic modulation effects. While crystalline sites exhibited a minimum ΔG_*H_ of 0.216 eV (HC3 configuration), amorphous domains demonstrated superior hydrogen adsorption energetics through three characteristic modes: 0.183 eV (HA3), 0.05 eV (HpA1), and 0.08 eV (HipA2). This marked enhancement originates from the disordered Ru arrangements that create metastable electronic configurations favoring bidirectional hydrogen intermediation. Regarding HzOR mechanisms, we mapped the complete dehydrogenation pathway involving sequential ^*^N_2_H_4_ → ^*^N_2_H_3_ → ^*^N_2_H_2_ → ^*^N_2_H → ^*^N_2_ transitions across eight catalytic sites (Figure 4c,d; Figure S30, Supporting Information). Kinetic analysis confirmed that the ^*^N_2_H_4_→^*^N_2_H_3_ step serves as the primary rate‐determining step (RDS) for most configurations (1.883 eV on crystalline Ru), consistent with prior reports.^[^ ^4^, ^20^, ^40^ ^]^ Notably, amorphous sites induced two critical modifications: 1) Reduced RDS energy barriers to 1.290 eV (HzA2 configuration) through enhanced N─H bond activation, and 2) emergence of alternative kinetic pathways where the RDS shifted to the ^*^N_2_H→^*^N_2_ step with remarkably low *ΔG* of 0.866 eV (HzipA1). These dual effects—thermodynamic barrier reduction and reaction pathway diversification—stem from the electronic heterogeneity inherent to amorphous domains. The charge density difference analysis at the crystalline‐amorphous interface was illustrated in Figure S31 (Supporting Information). It reveals intense and heterogeneous charge redistribution at the interface. Certain Ru sites exhibit charge accumulation whereas others show charge depletion, collectively establishing Ru sites with diverse electronic structures. Such diversity in electronic configurations facilitates the synchronous optimization of both proton‐coupled electron transfer (HER) and multi‐step dehydrogenation processes (HzOR), providing atomistic rationale for the observed bifunctional enhancement.

**Figure 5:**
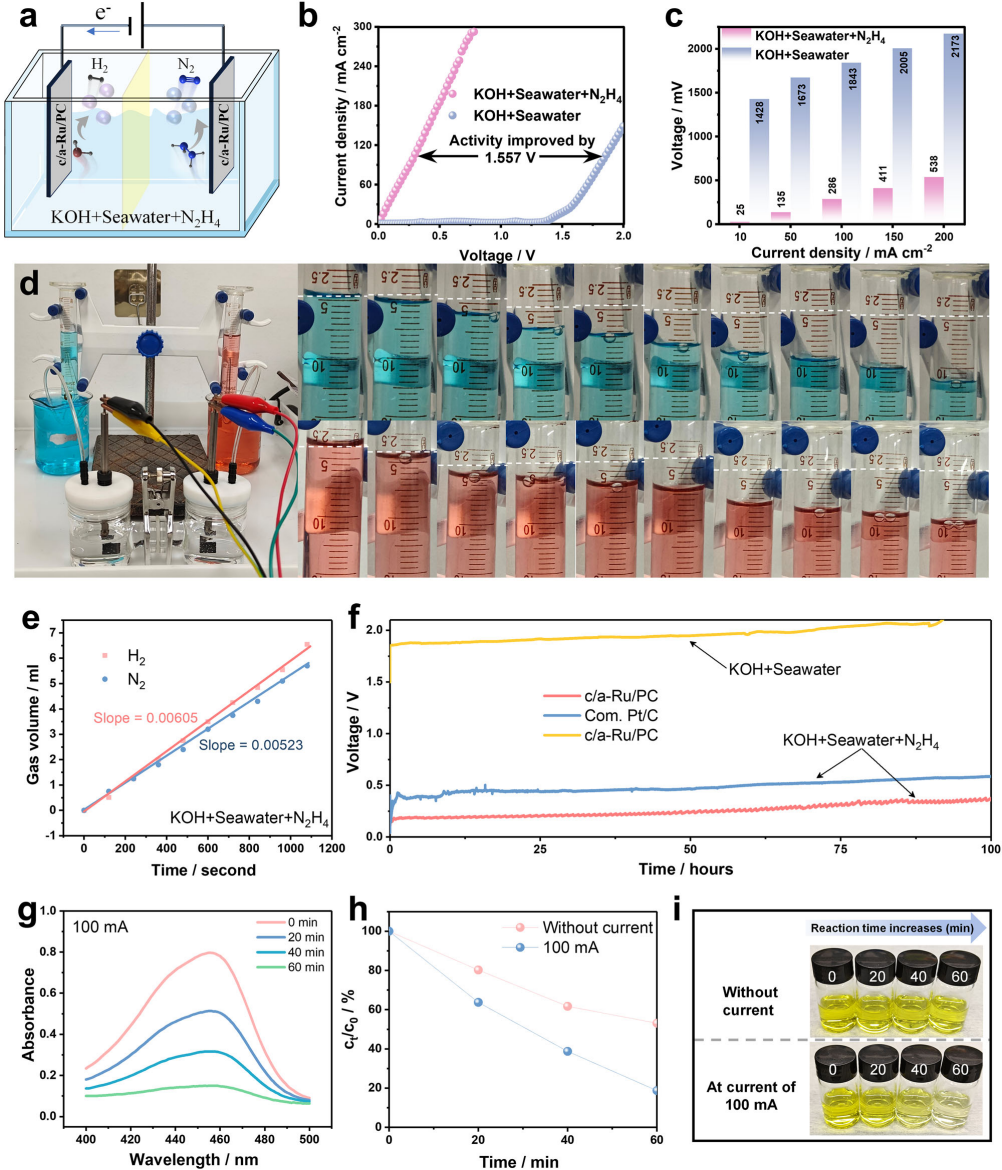
Two‐electrode electrolysis application. (a) Schematic diagram of c/a‐Ru/PC‐based electrolyzer. (b) LSV curves of c/a‐Ru/PC couple for OHzS and Pt/C‐IrO_2_ couple for overall seawater splitting (OWS). (c) Overpotential comparison of OHzS and OWS at different currents. (d) The gas collection device for OHzS in 1 m KOH + 1 m N_2_H_4_ + seawater solution, hydrogen, and nitrogen are indicated by blue and orange solutions, respectively. (e) The relationship between collection volume and time. (f) Chronopotentiometry curve for ca. 100 h at 20 mA cm^−2^. (g) UV–vis (UV) absorption spectra of N_2_H_4_ sewage degradation along with time at the current of 100 mA. (h) Percentage change in concentration for N_2_H_4_ sewage with and without applying current. (i) Optical image of colorimetric N_2_H_4_ assay after degradation by c/a‐Ru/PC catalyst with and without current.

Finally, the c/a‐Ru/PC catalyst enables energy‐efficient hydrogen generation via hydrazine‐assisted seawater splitting (OHzS) in a dual‐electrode alkaline system (**Figure** **5**a). The electrolyzer achieves a near‐zero onset voltage (0 V) and sustains a 1.557 V reduction in overpotential compared to conventional seawater splitting (OWS; Figure 5b,c). Real‐time gas quantification reveals H₂/N₂ evolution rates of 0.00605/0.00523 mL min⁻¹ (Figure 5d–f), deviating from the theoretical 2:1 ratio due to spontaneous N₂H₄ decomposition on c/a‐Ru/PC. Remarkably, the system maintains only slight voltage decay over 100 h (Figure 5g), demonstrating exceptional corrosion resistance. Hydrazine degradation assays (Figure 5h,i; Figure S32, Supporting Information) highlight c/a‐Ru/PC's dual functionality: 50% N_2_H_4_ removal within 1 h (0 mA) and >80% at 100 mA, eliminating toxic residues without auxiliary oxidants. Besides, the ammonia concentration measured after electrolysis at 100 mA for 1 h was 0.022 mmol L^−1^ (Figure S33, Supporting Information). This result confirms that less than 0.011% of hydrazine was decomposed into NH_3_. Thus, our material demonstrates exceptional selectivity toward catalytically converting hydrazine into environmentally benign nitrogen gas. This synergy of energy‐autonomous H₂ production and pollutant remediation positions c/a‐Ru/PC as a transformative solution for sustainable hydrogen economies.

## 3 Conclusion

By leveraging phosphorus‐mediated etching and doping, we engineered EMSI to construct semi‐crystalline Ru domains with metastable crystalline‐amorphous interfaces. The resulting catalyst exhibits exceptional bifunctional activity for HER (21.5 mV @ 10 mA cm⁻^2^) and HzOR (254 mV @ 10 mA cm⁻^2^), coupled with spontaneous hydrazine decomposition capability, enabling energy‐autonomous hydrogen production. Its operational robustness—retaining minimal performance decay under varying currents and temperatures—surpasses commercial Pt/C (20 wt.%). First‐principles computations rationalize these advances, revealing that amorphous Ru sites around the interface lowered the energy requirement for oxidation of adsorbed ^*^H and dehydrogenation from ^*^N_2_H_4_ to ^*^N_2_H_3_, together with RDS shift to the ^*^N_2_H→^*^N_2_ for accelerating bifunctional HER/HzOR electrocatalysis. This work establishes a paradigm for designing metastable electrocatalysts through EMSI‐driven atomic disorder, bridging structural hierarchy with sustainable energy conversion.

## Conflict of Interest

The authors declare no conflict of interest.

## Author Contributions

D.Y., R.Y., and H. Z. contributed equally to this work. D.Y. formal analysis, data curation, wrote the original draft and edited the final manuscript. conceptualization, investigation, methodology. R.Y. formal analysis, data curation, wrote the original draft. investigation, methodology. H.Z. formal analysis, data curation, wrote the original draft. investigation, methodology. J.L. data curation, investigation. S.S. investigation, methodology. Y.H. formal analysis, data curation, wrote the original draft and edited, supervision. Y.S. formal analysis, wrote the original draft and edited, supervision.

## Supporting information



Supporting Information

Supplemental Video 1

## Data Availability

The data that support the findings of this study are available from the corresponding author upon reasonable request.
