# Supplementary material for: Semi‐Crystalline Ruthenium Catalyst for Zero‐Drag Hydrogen Production from Hybrid Alkaline Seawater Electrolysis
Source: Adv Sci (Weinh). 2025 Jun 27;12(36):e07848. doi: 10.1002/advs.202507848 (PMC12462945; doi:10.1002/advs.202507848)
Supplement: Supplementary file 1 — Supporting Information [file ADVS-12-e07848-s001.docx]

**Semi-Crystalline Ruthenium Catalyst for Zero-Drag Hydrogen Production from Hybrid Alkaline Seawater Electrolysis**

Dongquan Yang^a,b,§^, Rui Yang^c,§^, Huayi Zeng^a,b,§^, Jiajun Luo^a,b^, Shuangjuan Shen^a,b^, Yiyin Huang^a,b,*^, Yuanyuan Sun^d,*^

^a^ *College of Physics and Energy, Fujian Normal University, Fujian Provincial Key Laboratory of Quantum Manipulation and New Energy Materials, Fuzhou, 350117, China*

^b^ *Fujian Provincial Engineering Technology Research Center of Solar Energy Conversion and Energy Storage, Fuzhou, 350117, China*

^c^ *School of Materials and Chemistry Anhui Agricultural University, Hefei, 230036, P.R. China*

*^d^ Institute of Innovation Materials and Energy, College of Chemistry & Chemical Engineering, Yangzhou University, Yangzhou 225002, China*

E-mail address: [hyy@fjnu.edu.cn](mailto:hyy@fjnu.edu.cn); syy@yzu.edu.cn

**Experimental Section**

*Chemical and reagents*

Pt/C (20wt.%Pt) from Johnson Matthey, potassium hydroxide (≥99%), hydrazine hydrate (85%), concentrated hydrochloric acid (37%), ethanol (99.7%), sodium borohydride (98%), ruthenium trichloride (≥35%Ru), ethylene glycol (99.5%), and Nafion solution (5wt.%) from Dupont were acquired and used directly in the synthesis. Hydrophilic carbon paper from Hesen was also used without additional treatment. Raw wood pulp paper was purchased from Vinda and immersed in ethanol and hydrochloric acid to remove any impurities. Argon (≥99.999%) was utilized during heating treatments. High-purity hydrogen gas (≥99.999%) was employed for degassing of dissolved oxygen in electrolyte before HER measurement.

*Synthesis of Catalysts*

For the synthesis of c/a-Ru/PC sample, 3 g raw wood pulp roll paper was immersed completely in a solution of 3 g sodium hypophosphite and 30 mL deionized water, then the mixture was put into a ventilated drying oven and kept at 120 ^o^C for drying. The dried mixture was then put in a tube furnace for thermal treatment at 1000 ^o^C under Ar atmosphere for 60 minutes. The resulting product was ultrasonically processed in 100 mL deionized water for 10min, then filtered, washed with deionized water several times, and dried in a ventilated drying oven at 60 ^o^C to obtain PC. 50 mg PC and specific amount of ruthenium trichloride solution (104.76 mM) were added into 50 mL ethylene glycol, following with ultrasonic treatment for 1 h. The mixture suffered from microwave processing at 800 W for 90 s. After cooling down to room temperature, the mixture was filtered, washed with deionized water several times, and dried to obtain c/a-Ru/PC-1000 material.

Controlled conditions for the synthesis of Ru/PC samples were conducted by changing the heat treatment temperature and the mass ratio of sodium hypophosphite to paper. In addition, the Ru/C and individual C samples were obtained in the same experimental procedure without addition of sodium hypophosphite, while the PC sample was acquired without loading of Ru. These controlled samples were tested by hydrogen evolution reaction in alkaline medium to optimize the synthesis condition.

*Physical characterization*

Transmission electron microscope (TEM, JEM-2100F) measurement was performed to observe the atomic/nano structures of the synthesized sample, and the built-in energy dispersion spectroscopy was used to determine the element dispersion of Ru, P, O, and C on the sample. Inductively-coupled plasma optical emission spectrometer (ICP-OES, Ultima2) and ion chromatograph were employed to analyse the Ru contents in the samples, and the (non)metal contents in seawater. X-ray powder diffractometer (Philip Xhilip Pro MPP) with a Cu Kα radiation (λ = 0.154 nm) was used to perform the X-ray powder diffraction (XRD) tests at 5° min^-1^. X-ray photoelectron spectroscopy (XPS) measurements were conducted at high vacuum condition (3×10^-10^ mbar) with C correction at the standard binding energy of 284.8 eV. An in-lens electron source and a low-energy Ar^+^ source were used to achieve the charge neutralization of the tested materials. Raman tests (excitation wavelength: 532 nm) was performed by using a Raman spectrometer of HR Evolution, HORIBA. A visible objective of 50× long working distance was used during Raman test and a silicon standard sample (520.7 cm^−1^) was employed for calibration. Temperature-programmed H_2_ desorption (H_2_-TPD) measurement was performed in a Microtrac BELCat II apparatus. Before test, the sample (60 mg) was treated at 300 °C under argon protection for 60 minutes. Then the sample was cooled down to room temperature and hydrogen flow was introduced for H_2_ adsorption. Afterwords, the argon flow (99.999%) was purged at 30 sccm for 1h. Finally, the sample was heated from 50 to 600 °C at 10 °C min^-1^ under Ar protection to track H_2_ desorption behaviour using a TCD detector. In-situ diffuse reflectance infrared Fourier transform spectra (in-situ DRIFTS) were recorded on the Bruker Vertex 70V Fourier transform infrared spectrometer equipped with a Mercury-Cadmium-Telluride (MCT) detector and a Harrick scientific DRIFT cell. Before test, the samples were reduced by H_2_ at 300 ℃ for 90 min, and then CO was injected into the samples for 60 min at room temperature, and then the adsorbed CO was desorbed by He flow purging.

Ru K-edge X-ray absorption spectroscopy (XAS) was obtained from the SPring-8 third-generation synchrotron radiation light source via testing in a transmission mode and employing the hard X-ray microanalysis (HXMA) beamline. The incident X-ray photons have an energy resolution of 2 × 10^−4^. The reference foils were used for calibration of the acquired spectra. The Athena software with the version of 0.9.26 was used for data analysis and processing, involving calibrations on pre-edge line and post-edge line from background. Fourier transformed fitting was performed using the Artemis software. The Ru foil extended X-ray absorption fine structure (EXAFS) data have provided the amplitude parameters. These parameters are crucial for data fitting and refining the coordination number (CN) of the absorption element, and kept constant during the fitting processes to supply the reliable input for precise refinement. A k^3^ weighting, k-range of 3 – ~14 Å^-1^, as well as an R range of 1–3 Å had been employed for fitting of Ru foil. For RuO_2_ and other materials, a k^3^ weighting, k-range of 3 – ~13.5 Å^-1^, and R range of 1 – 2 Å had been used. For analysis of Wavelet Transform, the χ(k) produced from Athena was imported into the Hama Fortran code according to the parameters as follows: k range of 0 – 16 Å^-1^, k weight of 2, R range of 0 - 4 Å, and a Morlet function with κ=10, σ=1 as the mother wavelet to supply the overall distribution.

*Electrochemical measurements*

Electrochemical measurements were mainly performed in a three-electrode electrolytic cell, which was connected to a CH Instrument Inc. (CHI 660e) electrochemical workstation. The electrolyte filled in the electrolytic cell was 1 M KOH solution or 1 M KOH + 1 M hydrazine solution. The three electrodes are a catalyst-loaded glassy carbon (GC, Ф = 4.0 mm) as working electrode, a graphite rod as counter electrode, and a Hg/HgO (1M KOH) or an Ag/AgCl electrode (sat. KCl) as the reference electrode. The preparation of working electrode was proceeded in the following procedure: a solution consisted of 950 μL isopropanol/50 μL Nafion solution (5%) was used to disperse 10 mg of the as-prepared material (or commercial Pt/C) under ultrasonication. 4 μL of the catalyst ink was then pipetted and coated on the polished GC electrode, following by drying naturally. The loading of catalyst on GC is 32 μg cm^−2^.

Steady-state linear sweep voltammetry (LSV) curves for HER and hydrazine oxidation reaction (HzOR) were recorded at the scan rate of 10 mV s^-1^. Electrochemical AC impedance spectra (EIS) was recorded in the frequency range of 100,000 to 0.01 Hz at a potential amplitude of 5 mV. Cyclic voltammetry (CV) curves are recorded in the non-Faraday potential range to evaluate the electrical double-layer capacitance (C_dl_). Open circuit potential experiment was carried out to analyse the amount of adsorbed O-containing species on catalyst surface. The potentials recorded in this work were further converted into the standard electrode potentials *vs.* the reversible hydrogen electrode (RHE) according to the potential calibration by CV method, which was illustrated in Figure S11.

The hybrid seawater electrolysis measurements were performed in a custom-made electrolysis cell, in which the cathode and anode were consisted of c/a-Ru/PC/Ni foam (1 mg cm^-2^), with a 30 µm-thickness anion-exchange membrane (fumasep FKS-75),) in-between. The Ni foam (0.2 mm thickness) was used as the current collectors. The electrolyte employs 1 M KOH or 1 M KOH + N_2_H_4_ solution with the flow rate of 5 ml min^−1^ for circulating in the two chambers. The catalyst loading on the Ni foam was fixed at 1 mg cm^-2^. All the voltages in the electrolytic cell measurements were recorded at ambient pressure and room temperature.

*Ammonia and hydrazine content analysis*

Ammonia concentration in the electrolyzed test solutions was quantified using the indophenol blue method. ‌Residual hydrazine was decomposed over copper plates prior to adding the color reagent. For each analysis, 1 mL of the post-electrolysis electrolyte was mixed with 1 mL of a NaOH/salicylic acid/sodium citrate solution (5 wt% each component). Subsequently, 0.5 mL of 0.05 M sodium hypochlorite and 0.1 mL of sodium nitroprusside solution (1 wt.%) were added. UV-vis absorption spectra were recorded, with absorbance measured at 655 nm. A calibration curve was generated using standard ammonium chloride solutions.

Hydrazine concentration was assessed via the Watt and Chrisp method. A color reagent was prepared by dissolving p-dimethylaminobenzaldehyde (0.6 g) in a mixture of concentrated HCl (12 M, 3 mL) and ethanol (30 mL). For analysis, 2 mL of the post-electrolysis electrolyte was combined with 2 mL of this reagent. Absorbance measurements were performed at 455 nm.

*Computational details*

The density functional theory (DFT) framework, employing the projected augmented wave (PAW) approach,^[1]^ was utilized for all computational simulations. These calculations were executed via the Quantum ESPRESSO software package,^[2]^ with the generalized gradient approximation (GGA) parameterized by the Perdew-Burke-Ernzerhof (PBE) functional^[3]^ to model exchange-correlation interactions. A plane-wave basis set was adopted to expand the Kohn-Sham orbitals, applying a kinetic energy cutoff threshold of 450 eV. The system was modeled using a supercell of dimensions 8.12 Å × 12.84 Å × 26.55 Å, incorporating a vertical vacuum layer exceeding 15 Å along the *z*-axis to eliminate periodic boundary interference. For Brillouin zone integration, a Monkhorst-Pack grid^[4]^ with 3 × 3 × 1 k-point sampling density was implemented. Structural relaxation procedures constrained atomic positions in the substrate's basal layer while enforcing an energy convergence criterion of 1×10^−4^ eV.^[4]^ Intermediate species' Gibbs free energies were derived through the established Nørskov computational framework.^[5]^

The free energy change (∆G_H_) for hydrogen adsorption on the metal surface during hydrogen evolution reaction (HER) was calculated as:

∆G_H_ = ∆E_H*_ + ∆ZPE - T∆S (*Eq.* 1)

where ∆E_H*_, ∆ZPE and ∆S are the binding energy of hydrogen on the Ru surface, the change of zero-point energy (ZPE), the entropy change, respectively.

Six consecutive elementary steps were involved in the oxidation of hydrazine to nitrogen and hydrogen proceeds through:

(A) * + N_2_H_4_ → *N_2_H_4_ (*Eq.* 2)

(B) *N_2_H_4_ → *N_2_H_3_ + H^+^ + e^-^ (*Eq.* 3)

(C) *N_2_H_3_ → *N_2_H_2_ + H^+^ + e^-^ (*Eq.* 4)

(D) *N_2_H_2_ → *N_2_H + H^+^ + e^-^ (*Eq.* 5)

(E) *N_2_H → *N_2_ + H^+^ + e^-^ (*Eq.* 6)

(F) *N_2_ → N_2_ + * (*Eq.* 7)

where * represents a clean surface, while _*_N_2_H_4_, _*_N_2_H_3_, _*_N_2_H_2_, _*_N_2_H, and _*_N_2_ denote surfaces adsorbed with the corresponding species. The relative reaction energies can be calculated as:

∆E_A_ = ∆E_*N2H4_ – ∆E_*_ – ∆E_N2H4_ (*Eq.* 8)

∆E_B_ = ∆E_*N2H3_ + 0.5E_H2_ – ∆E_*N2H4_ (*Eq.* 9)

∆E_C_ = ∆E_*N2H2_ + 0.5E_H2_ – ∆E_*N2H3_ (*Eq.* 10)

∆E_D_ = ∆E_*N2H_ + 0.5E_H2_ – ∆E_*N2H2_ (*Eq.* 11)

∆E_E_ = ∆E_*N2_ + 0.5E_H2_ – ∆E_*N2H_ (*Eq.* 12)

∆E_F_ = ∆E_*_ + E_N2_ – ∆E_*N2_  (*Eq.* 13)


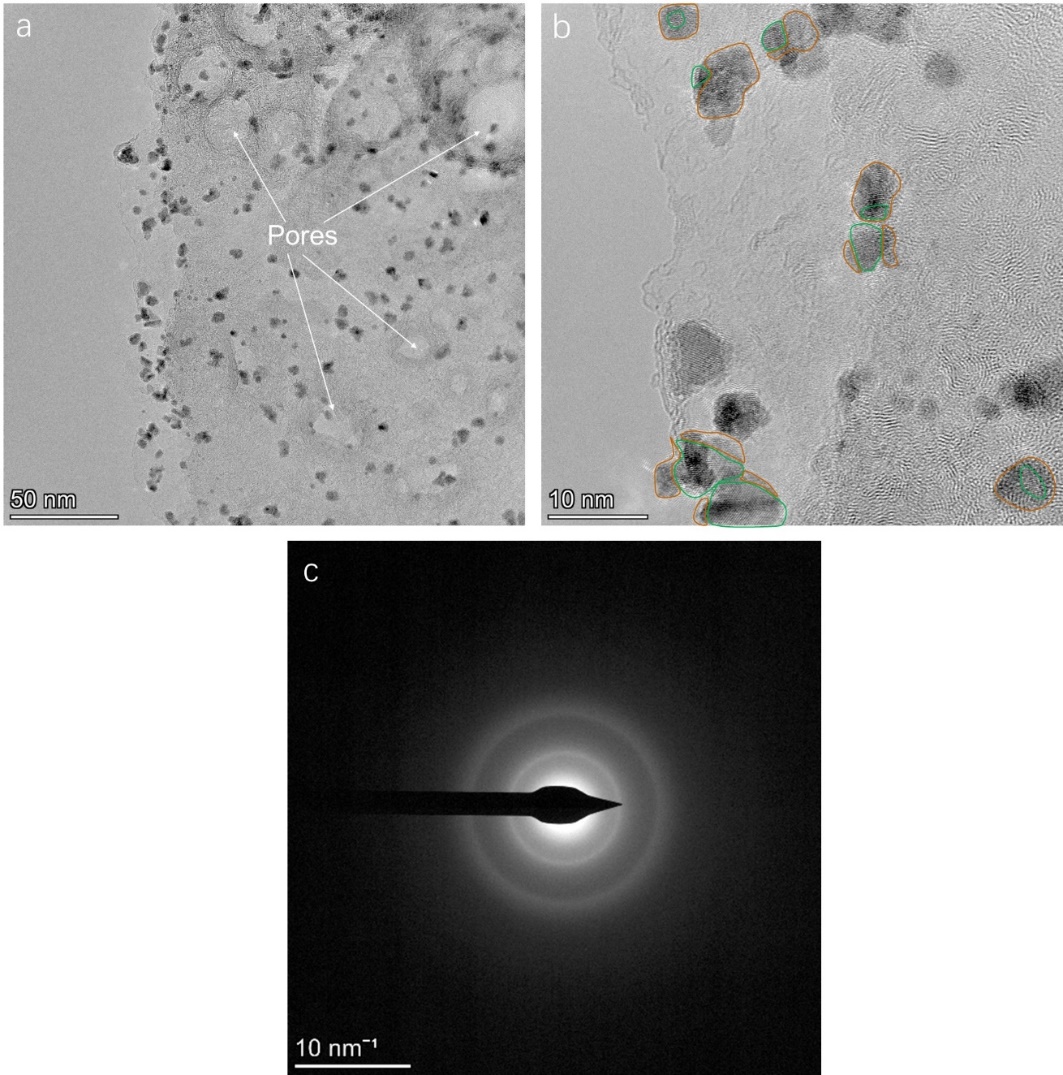


**Figure 1.** (a) Low-magnification and (b) high-magnification transmission electron microscopy (TEM) images for c/a-Ru/PC material. The amorphous regions were labeled by the orange circles, while the crystalline regions were labeled by the green circles. (c) Selected area electron diffraction (SAED) measurement for c/a-Ru/PC material.


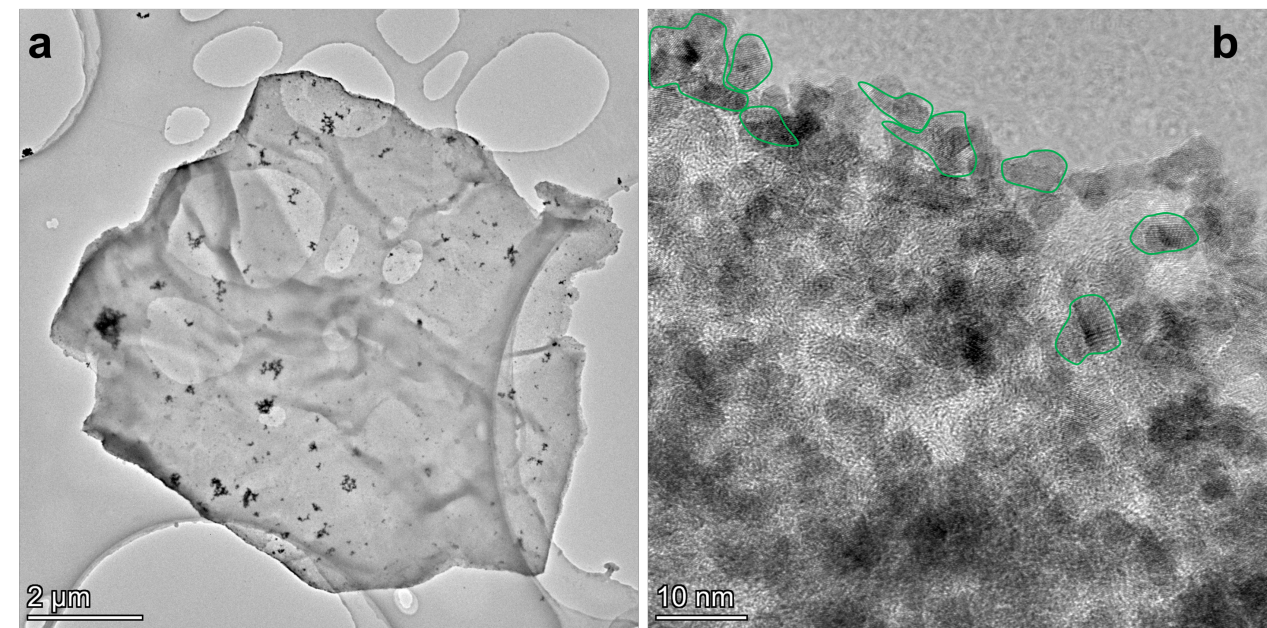


**Figure 2.** (a) Low-magnification TEM image and (b) high-magnification TEM image of Ru/C sample. It can be found that nanoparticles were agglomerate, and abundant crystalline regions were presented as labeled by green circles.


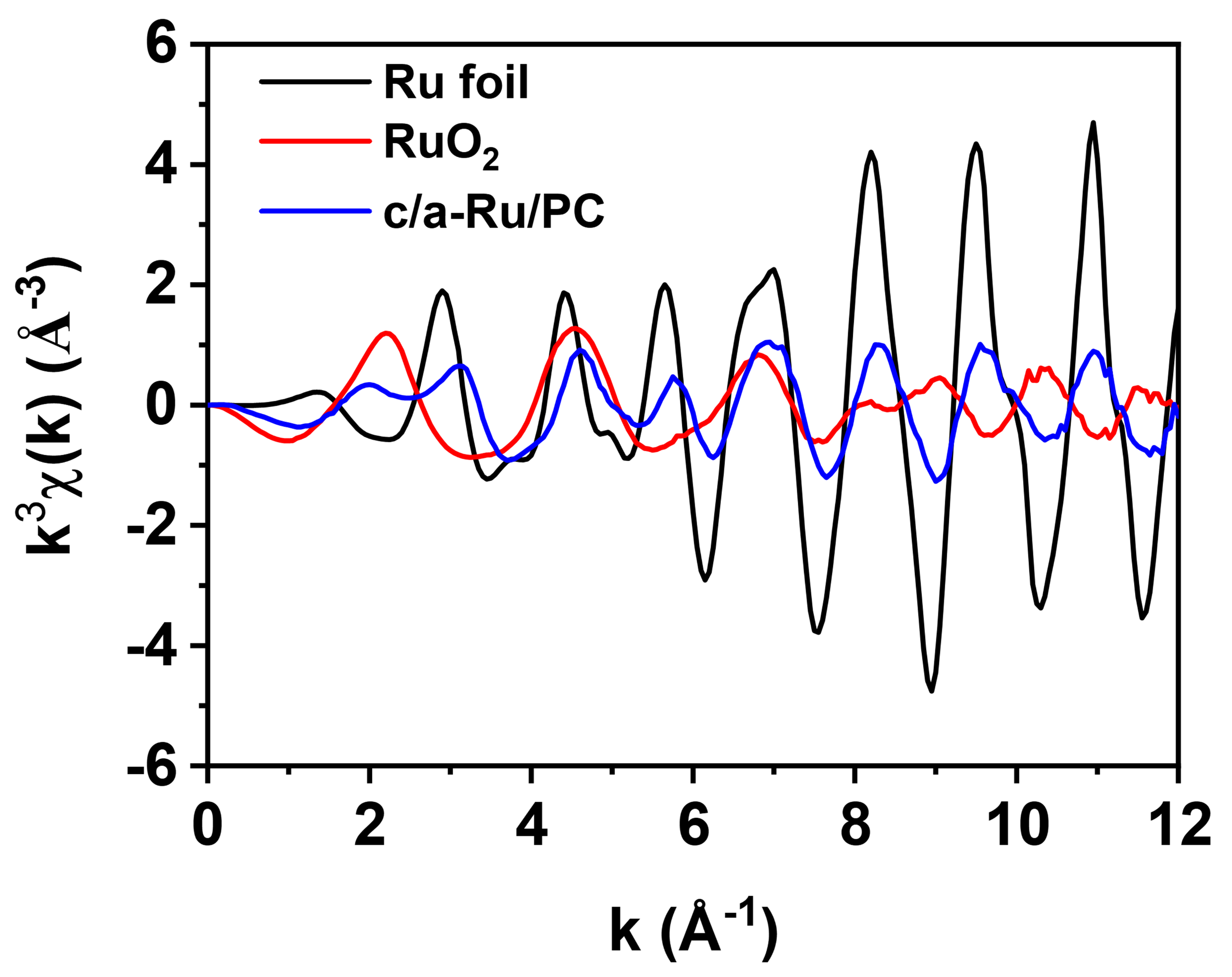


**Figure 3.** Ru K-edge k space curves of Ru foil, RuO_2_, and c/a-Ru/PC materials.


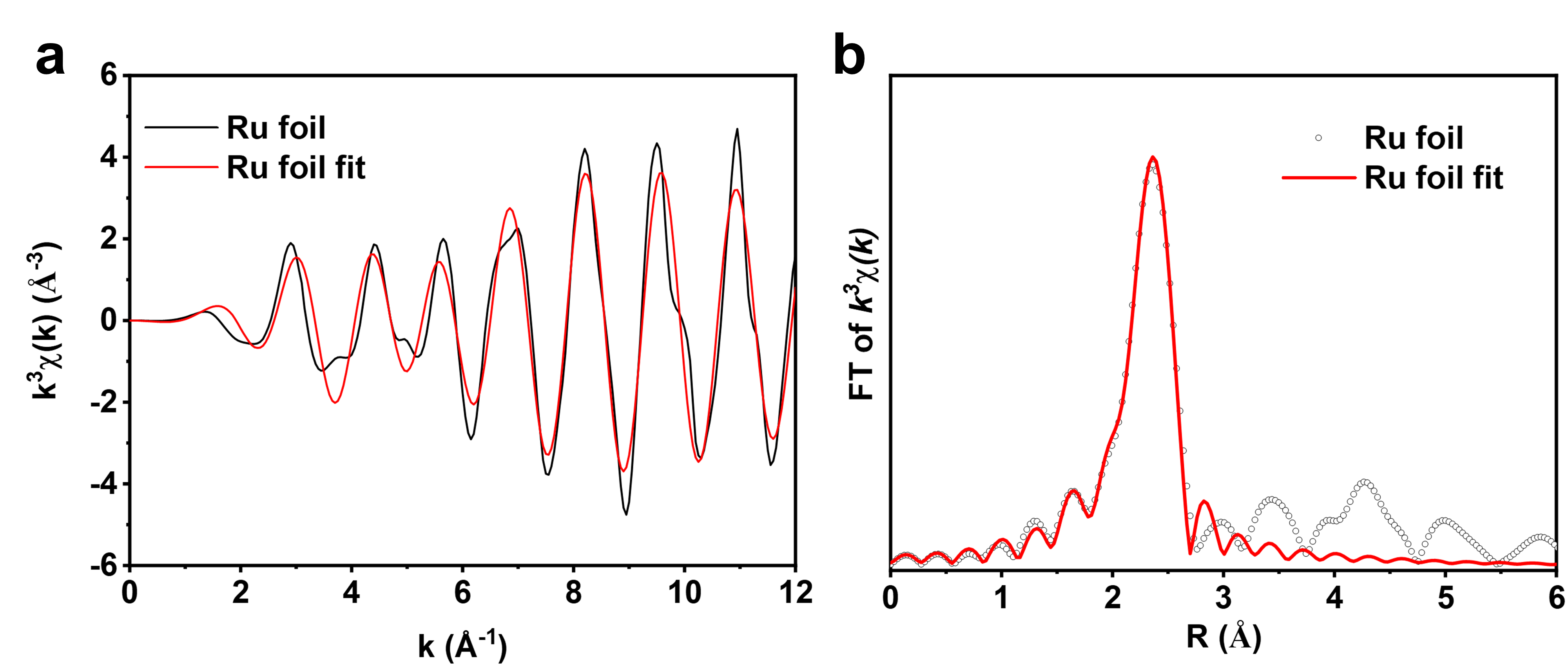


**Figure 4.** Ru K-edge (a) k and (b) R spaces fitting curves of Ru foil material.

**
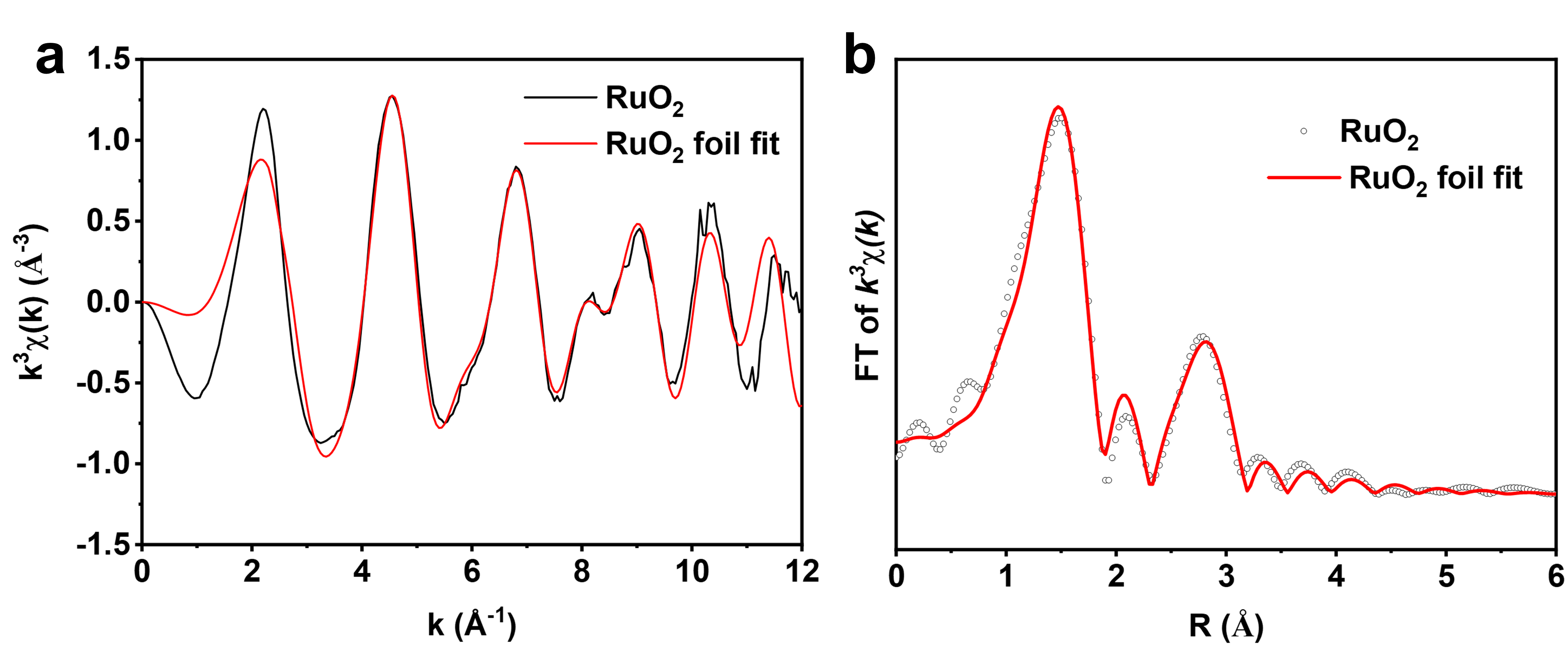
**

**Figure 5.** Ru K-edge (a) k and (b) R spaces fitting curves of RuO_2_ material.


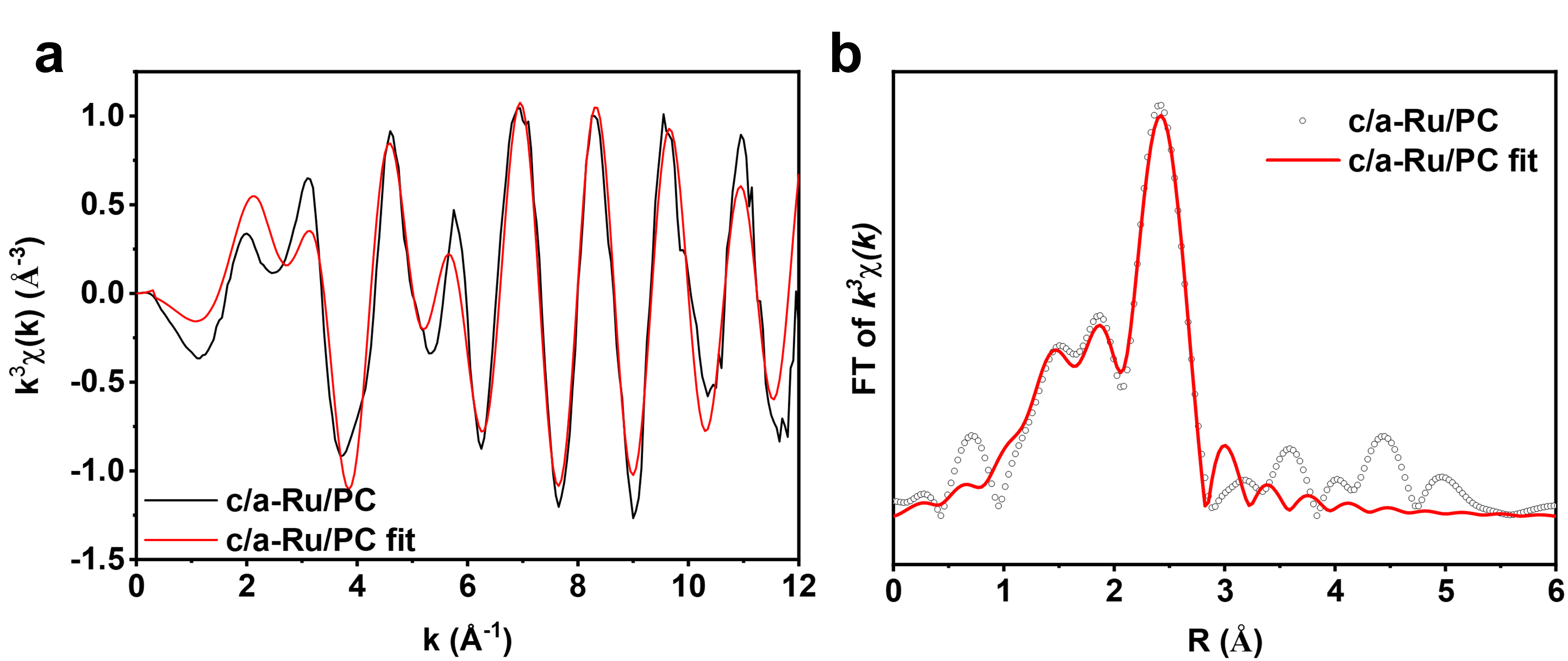


**Figure 6.** Ru K-edge (a) k and (b) R spaces fitting curves of c/a-Ru/PC material.


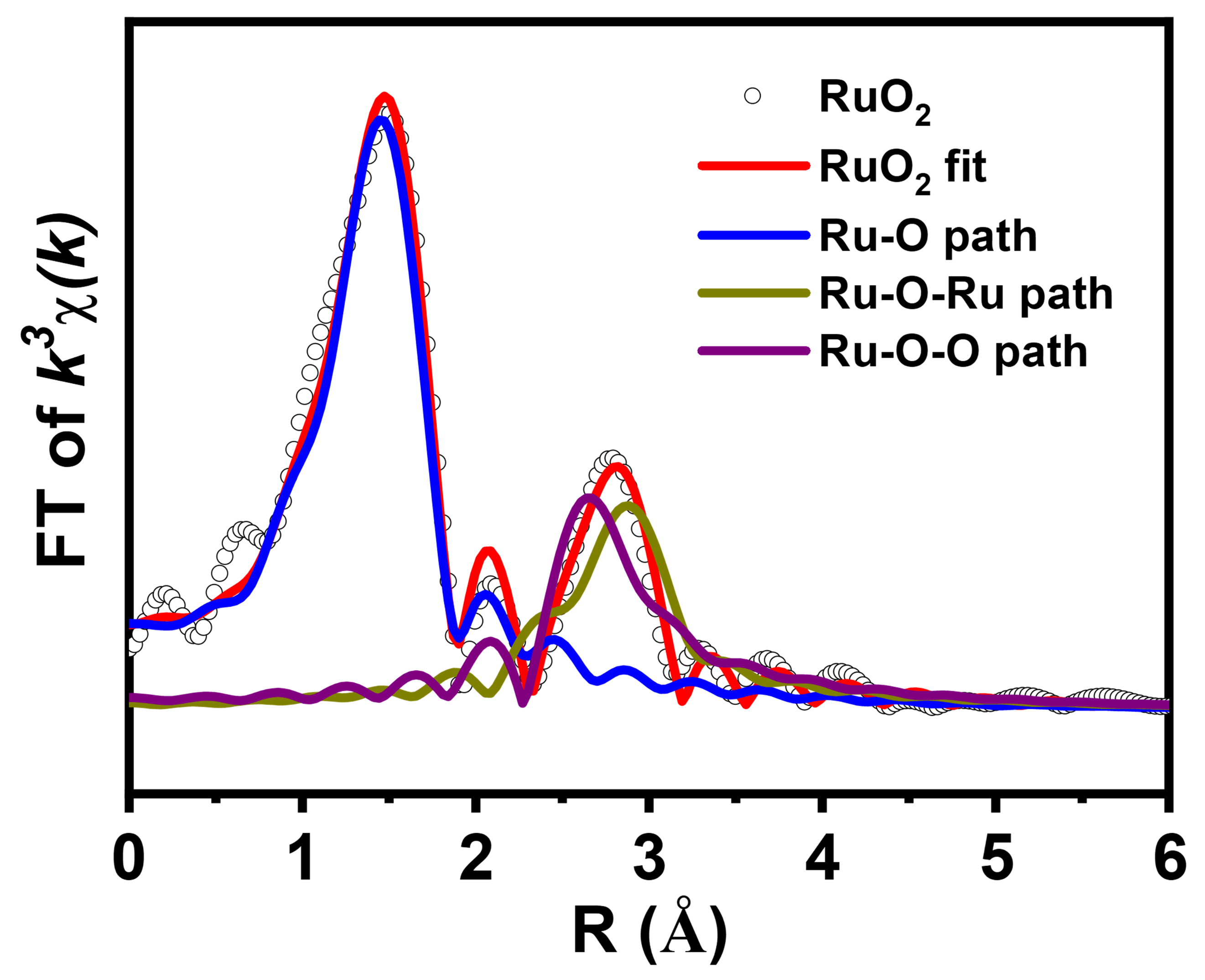


**Figure 7.** Ru K-edge R space fitting curve and the corresponding paths of RuO_2_ material.


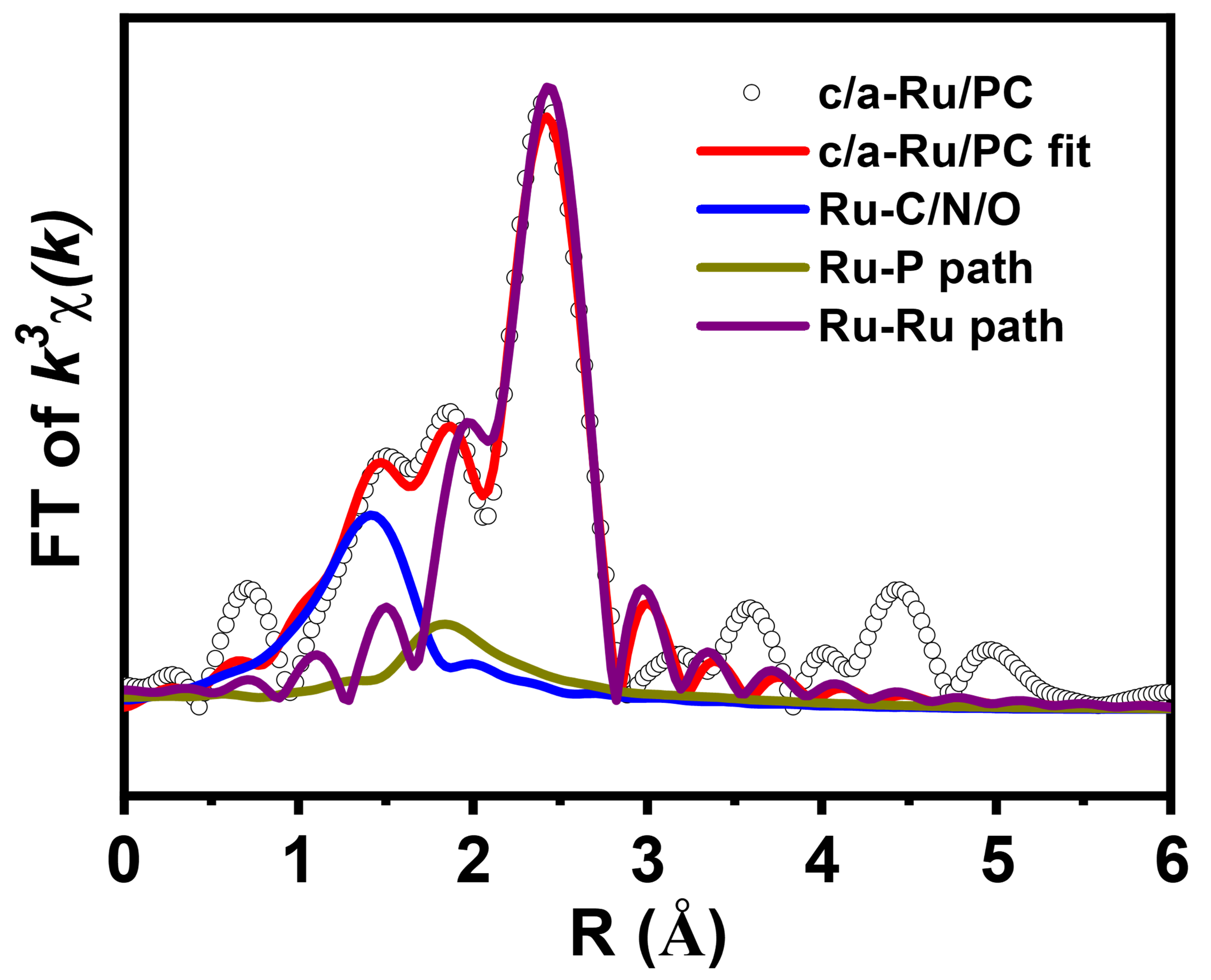


**Figure 8.** Ru K-edge R space fitting curve and the corresponding paths of c/a-Ru/PC material.


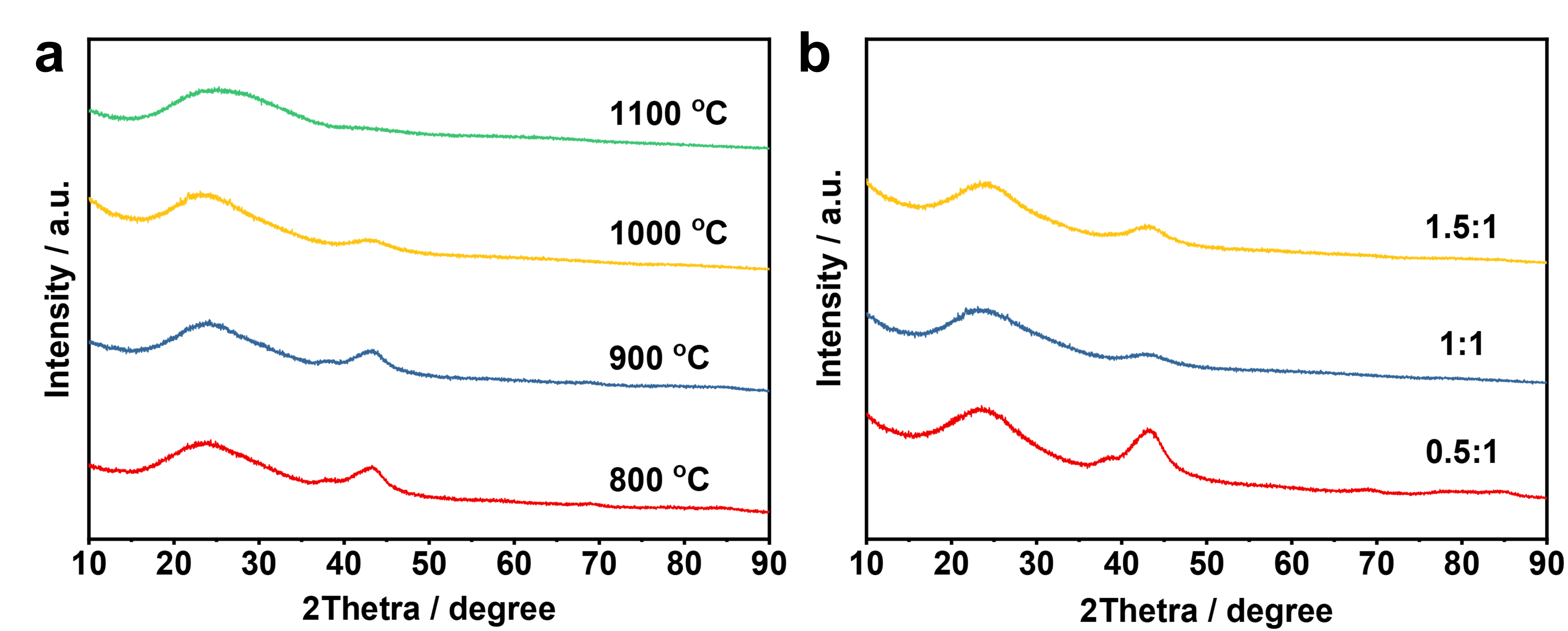


**Figure 9.** X-ray powder diffraction (XRD) patterns of the c/a-Ru/PC samples prepared at (a) different pyrolysis temperature and (b) different mass ratio of sodium hypophosphite/paper.


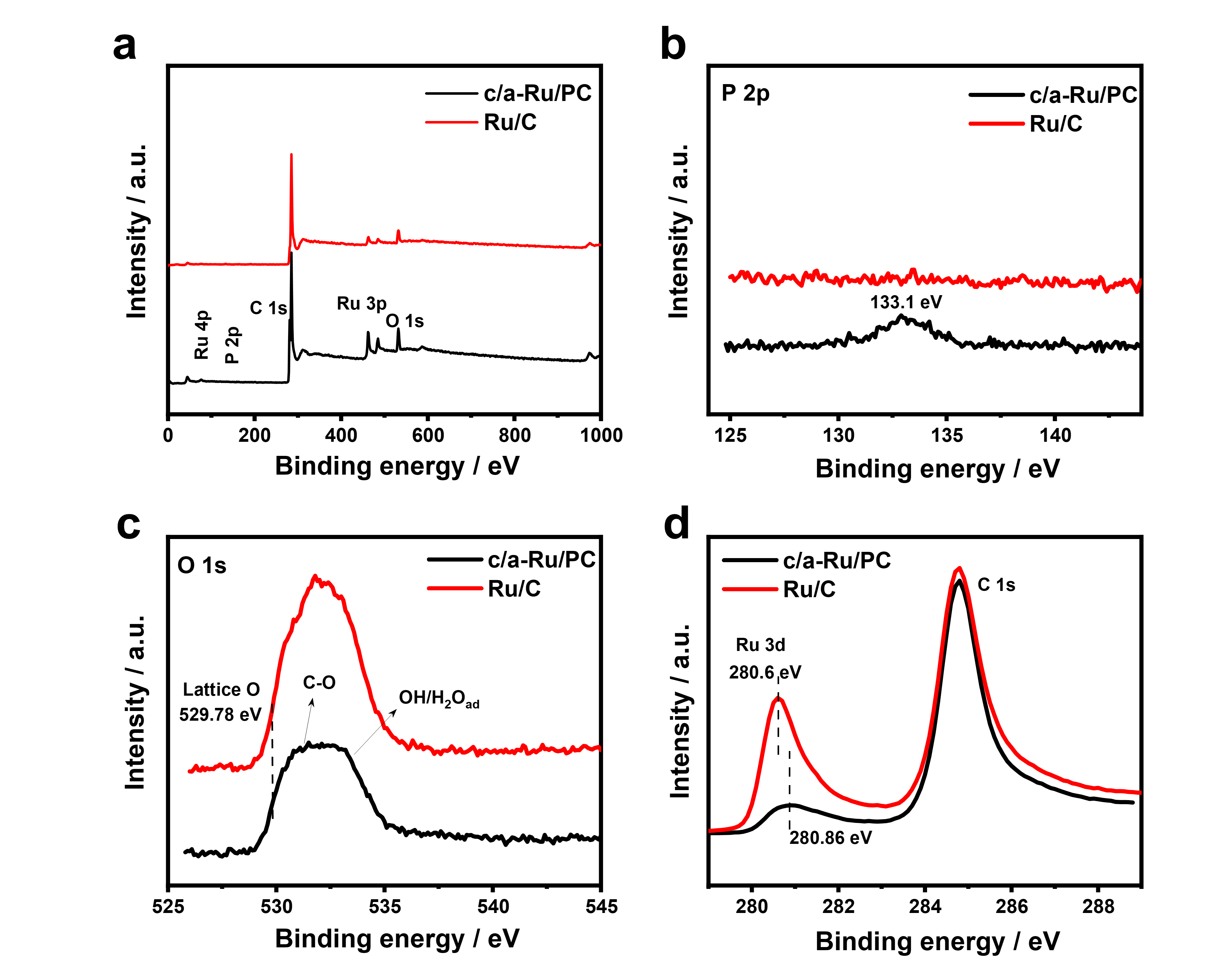


**Figure 10.** (a) Survey of X-ray photoelectron spectroscopy (XPS) spectra for the as-prepared c/a-Ru/PC and Ru/C materials. High-resolution XPS spectra of (b) P 2p, (c) O 1s, and (d) C 1s for c/a-Ru/PC and Ru/C materials.


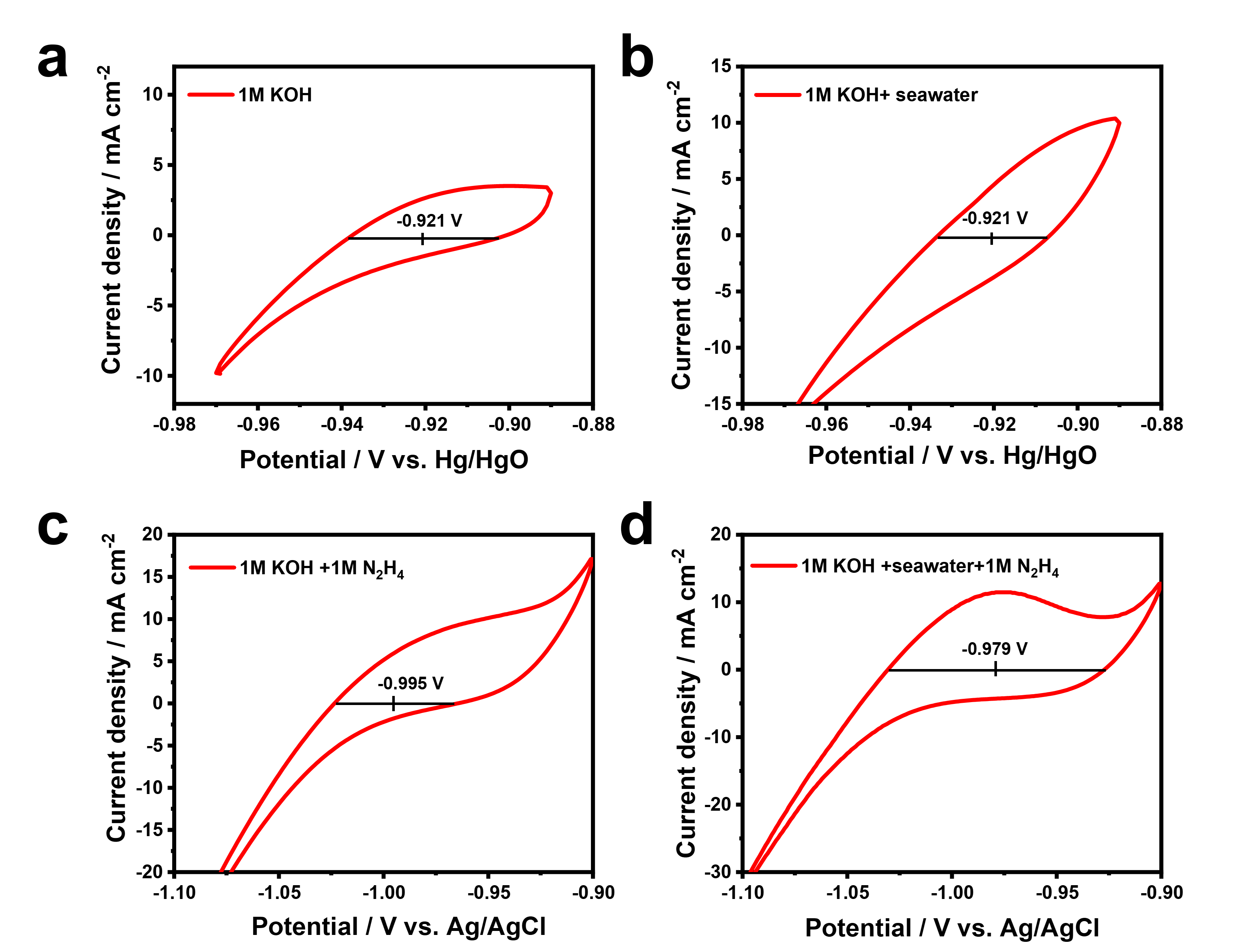


**Figure 11.** Potential calibration of the reference electrode in different solutions.

The Hg/HgO(1M KOH) or Ag/AgCl/KCl (sat.) electrode was used as the reference electrode for the measurements in reversible hydrogen electrode (RHE) calibration. The working electrode employs a Pt plate, and the solution was saturated by hydrogen. The cyclic voltammetry (CV) was conducted and the average value of anode and cathode potentials at the current of 0 was used to be the thermodynamic potential for the hydrogen electrode reactions.

E = E(Hg/HgO) + 0.921 V (RHE in 1 M KOH, 1M KOH+seawater)

E = E(Ag/AgCl) + 0.995 V (RHE in 1M KOH+1M N_2_H_4_)

E = E(Ag/AgCl) + 0.979 V (RHE in 1M KOH+seawater+1M N_2_H_4_)


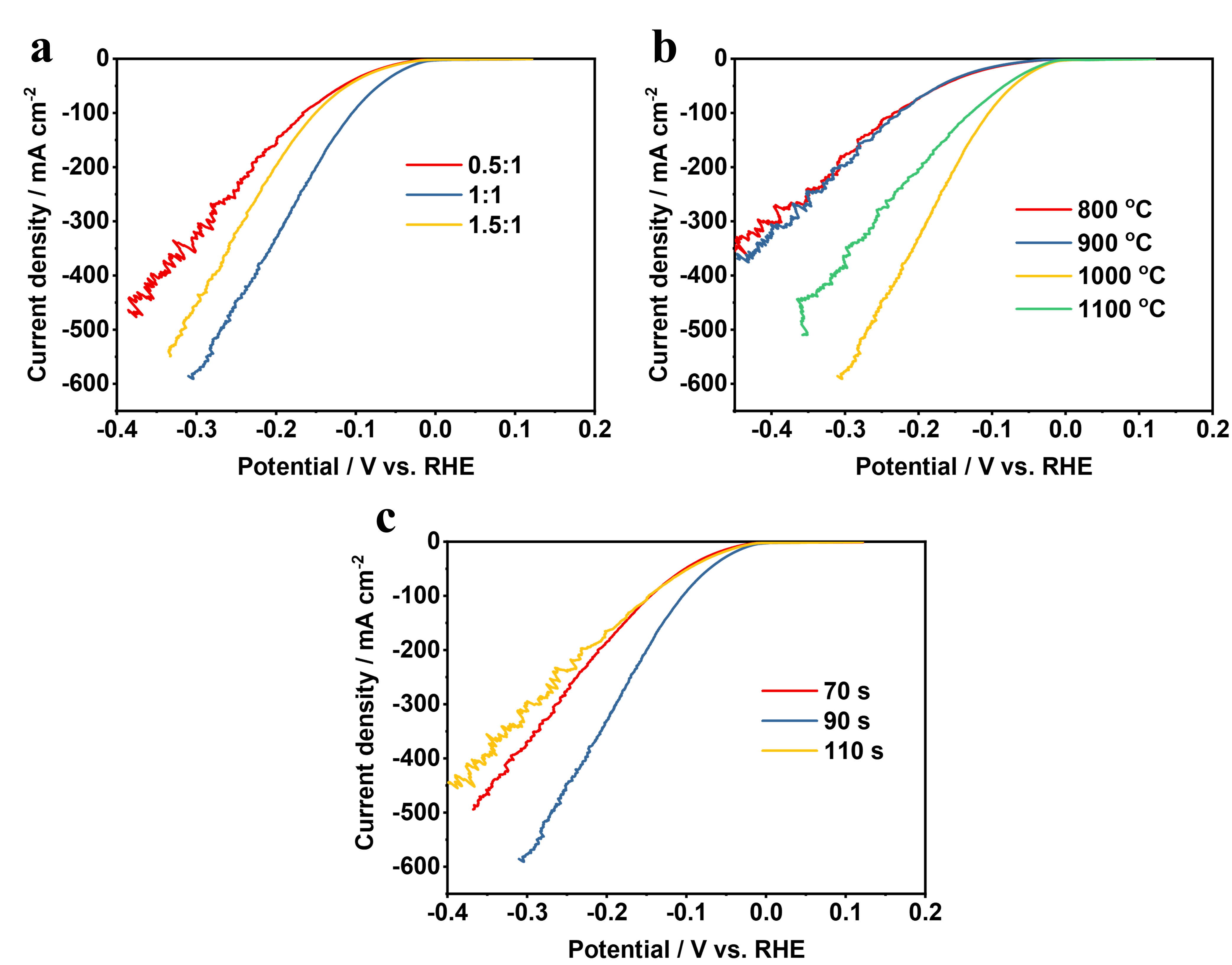


**Figure 12.** HER linear sweep voltammetry (LSV) curves of c/a-Ru/PC catalysts prepared at different (a) mass ratio of sodium hypophosphite/paper, (b) pyrolysis temperature of carbon, and (c) microwave time.


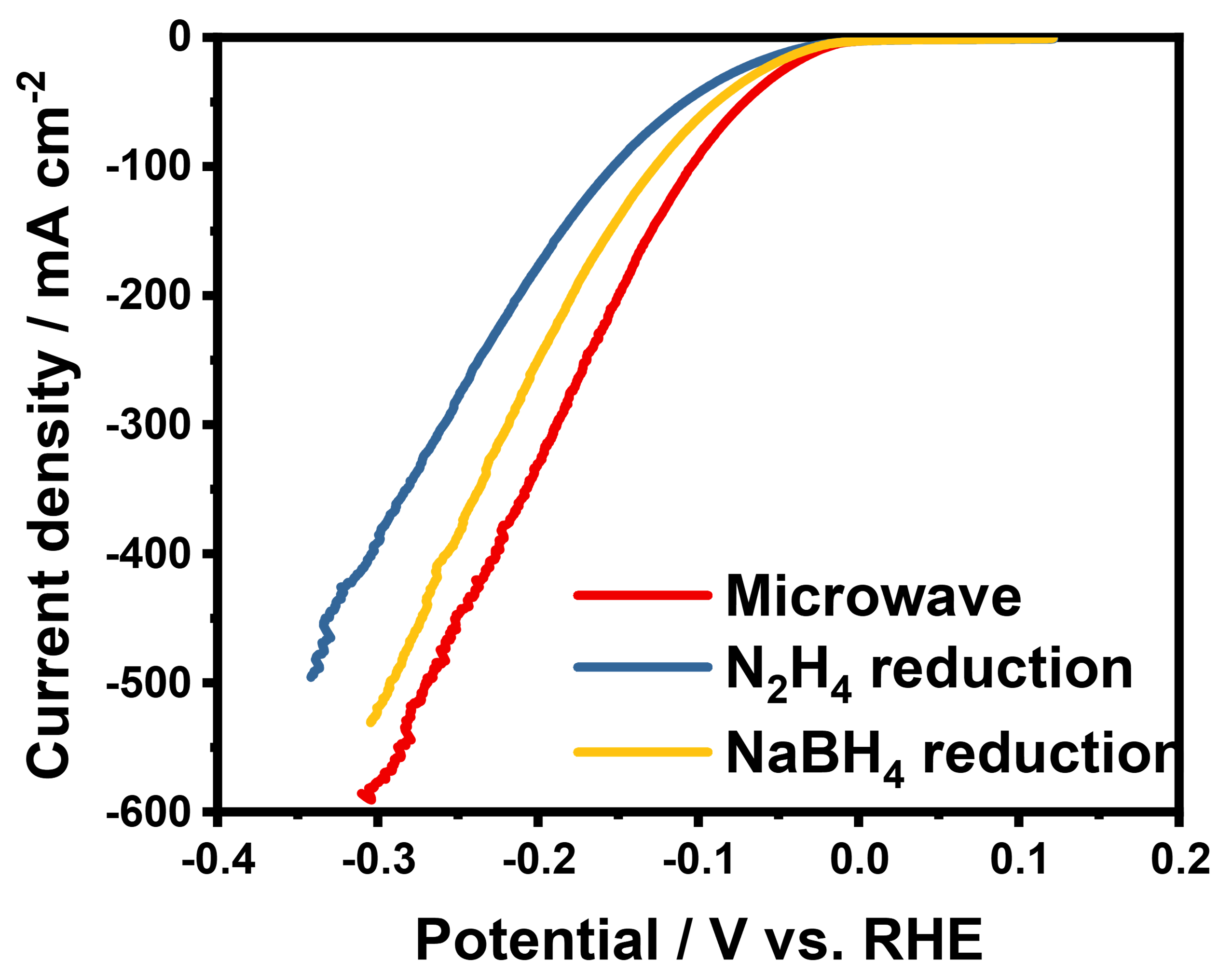


**Figure 13.** HER LSV curves of c/a-Ru/PC catalysts prepared by different methods.


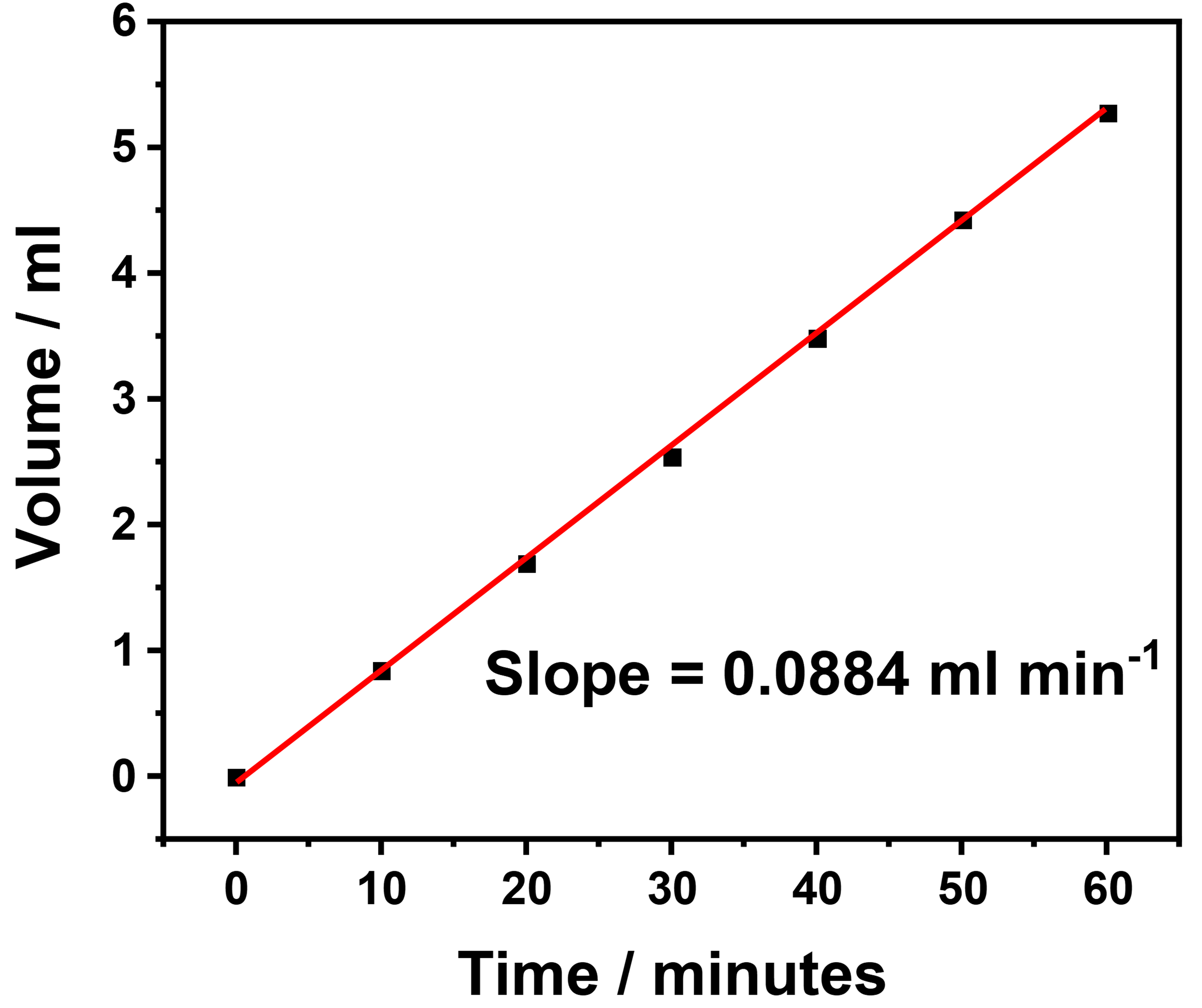


**Figure 14.** Faradaic efficiency for H_2_ production of the c/a-Ru/PC electrode in the 1 M KOH electrolyte. The average value of two independent experiments was used as the data in the figure.


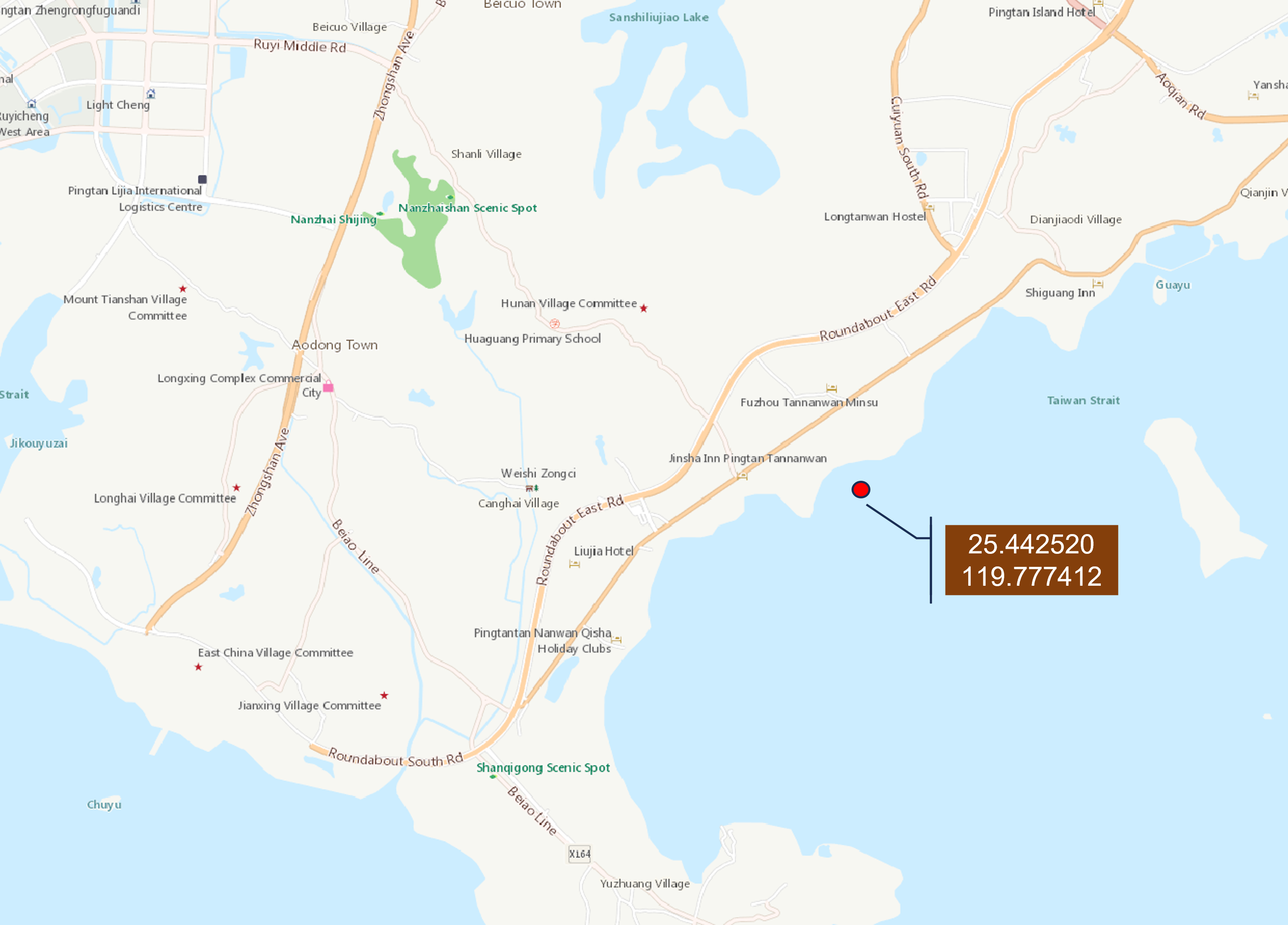


**Figure 15.** The location of sampling seawater for measurement in this study. Before test, the seawater was filtered through porous membrane (0.22 μm) to remove the particulate matter.


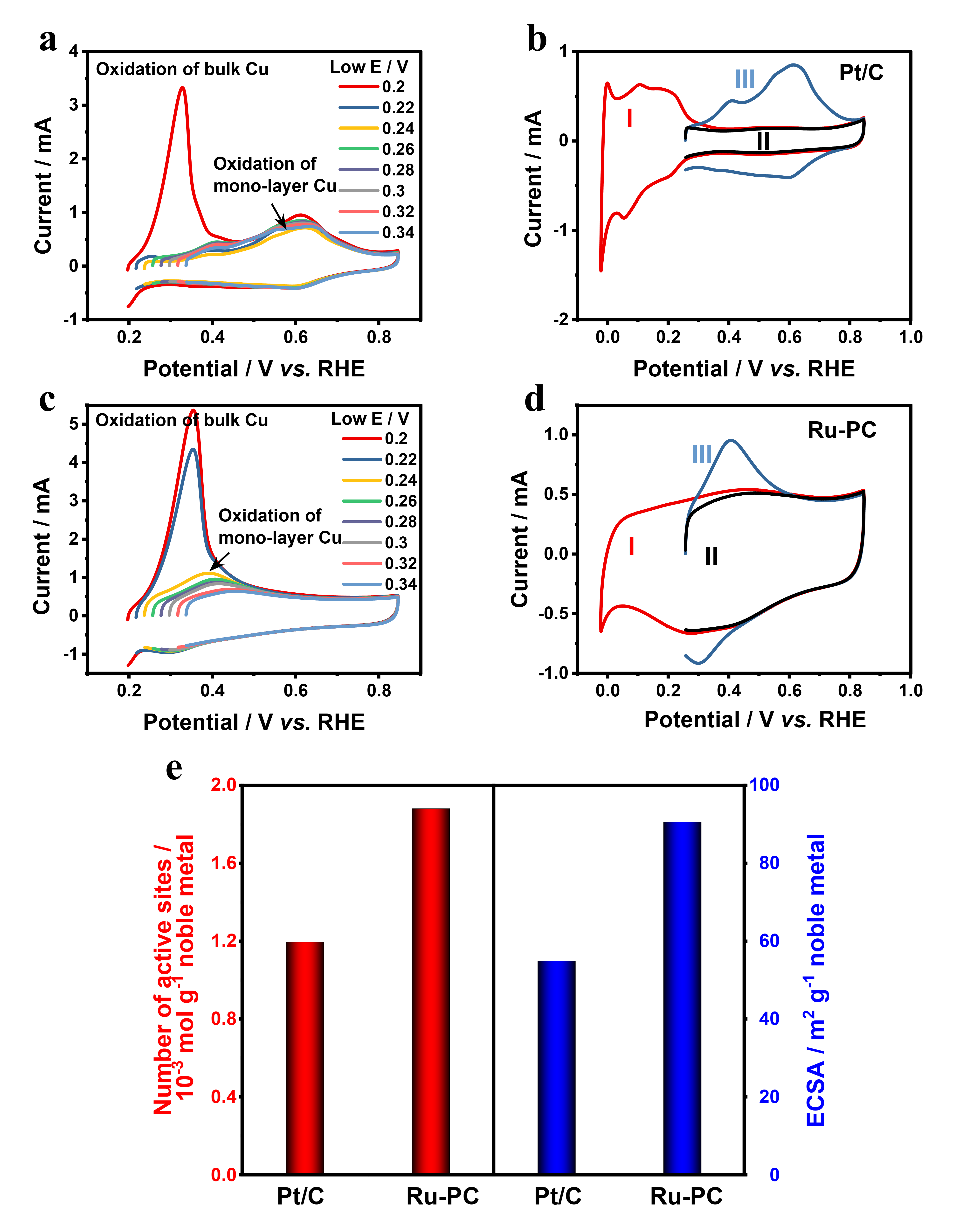


**Figure 16.** Copper underpotential deposition (Cu-UPD) test for evaluation of the number of active sites and electrochemical active area (ECSA). Cu-UPD in 0.5 M H_2_SO_4_ in the presence of 5 mM CuSO_4_ on (a) Pt/C and (c) Ru-PC with the electrode polarization at 0.2, 0.22, 0.24, 0.26, 0.28, 0.3, 0.32, and 0.34 V for 100 s, respectively, to evaluate the formation of UPD layer. Cu-UPD on (b) Pt/C and (d) Ru-PC in 0.5 M H_2_SO_4_ (I) without or (III) with 5 mM CuSO_4_ in the solution. The II curve is the background LSV from the onset potential same as III curve. Scan rate: 100 mV s^-1^. When the electrode was polarized at 0.26V for 100 s, only one anode peak ascribed to Cu oxidation was observed, attributing to the underpotentially deposited monolayer copper. Thus, the polarized potential was set at 0.26 V to obtain a monolayer copper for calculations of active sites and ECSA.

For active sites calculation (n): the UPD copper stripping charge was first obtained from the integrated area. The number of active sites was then calculated according to this equation: n=Q_Cu_/2F, in which Q_Cu_ is the integrated charge, and F is the Faraday constant (96484.5 C mol^−1^). For ECSA calculation, the equation was applied: ECSA = Q_Cu_/0.42, in which 0.42 represents the charge requested for monolayer copper adsorption.

The number of active sites was further used for calculation of the turnover frequency (TOF (s^-1^)) according to the equation: TOF=I/(2Fn) for HER, and TOF=I/(4Fn) for HzOR, in which I represents the current (A) during electrochemical tests.


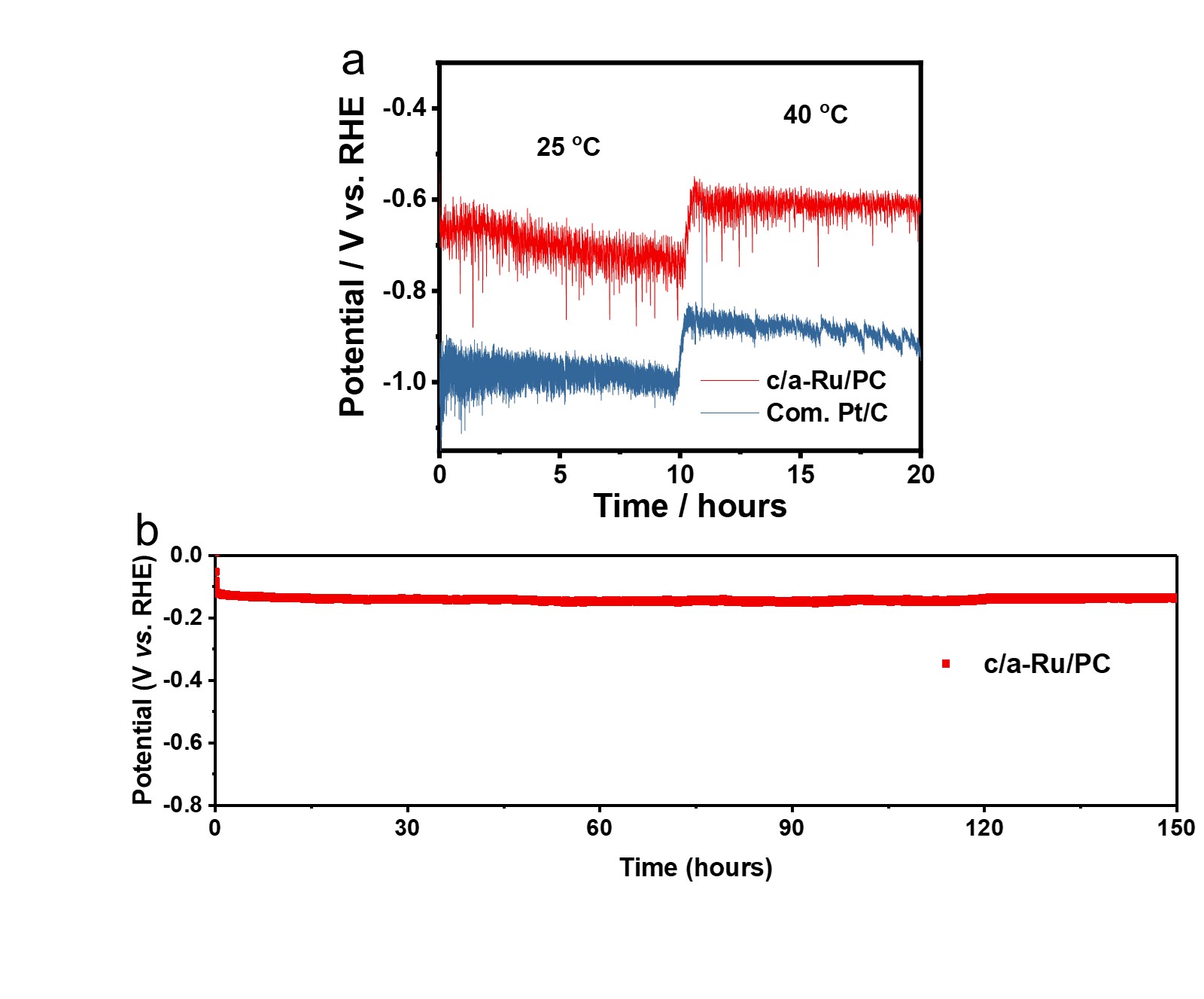


**Figure 17.** (a) HER stability test by chronopotentiometry of c/a-Ru/PC and commercial Pt/C catalysts at different temperature in 1 M KOH + seawater solution. (b) 150 hours’ HER stability test of c/a-Ru/PC at 20 mA cm^-2^.


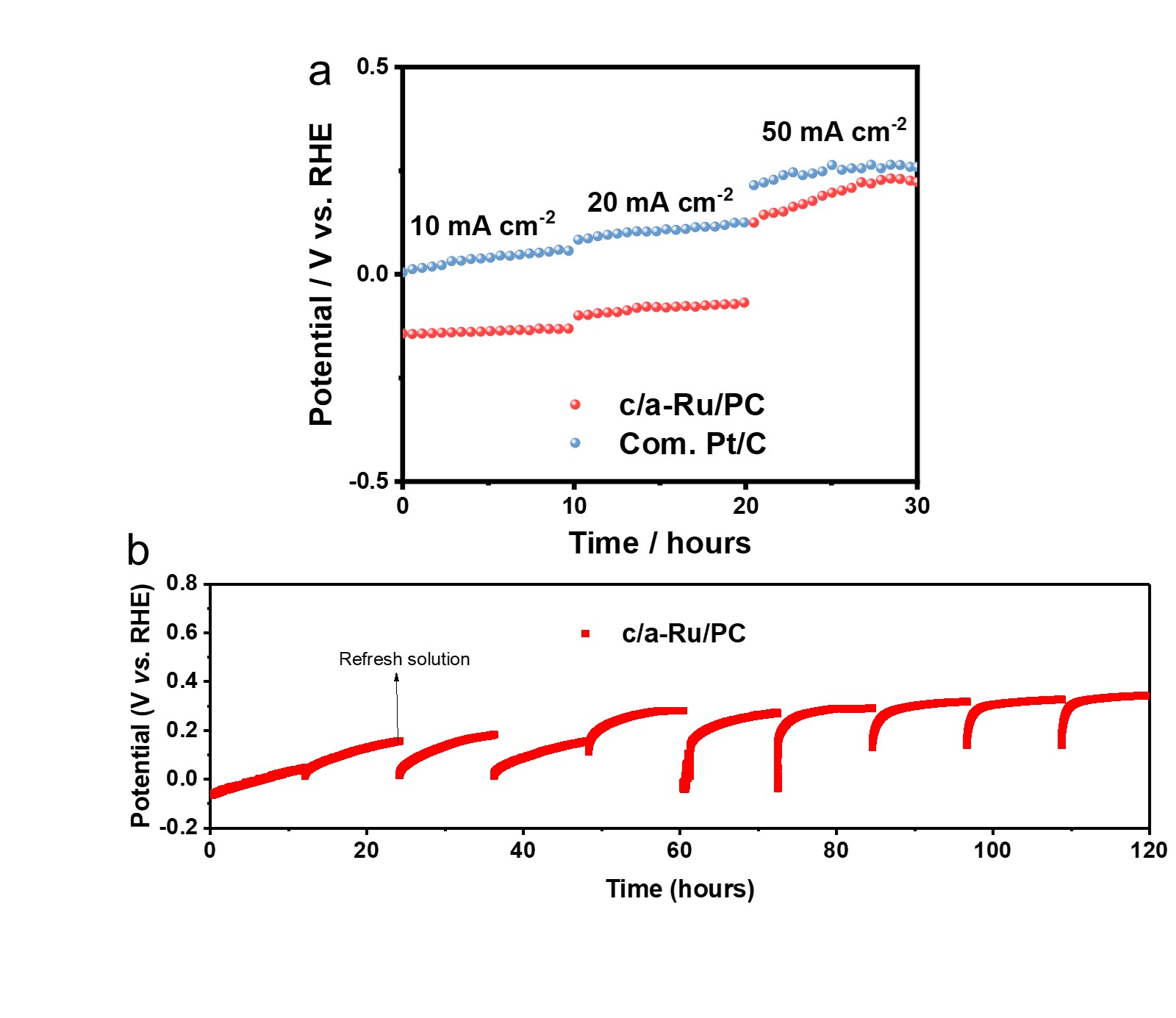


**Figure 18.** (a) HzOR stability test by chronopotentiometry of c/a-Ru/PC and commercial Pt/C catalysts at different currents in 1 M KOH + 1 M N_2_H_4_ solution. (b) 120 hours’ HzOR stability test of c/a-Ru/PC at 20 mA cm^-2^.


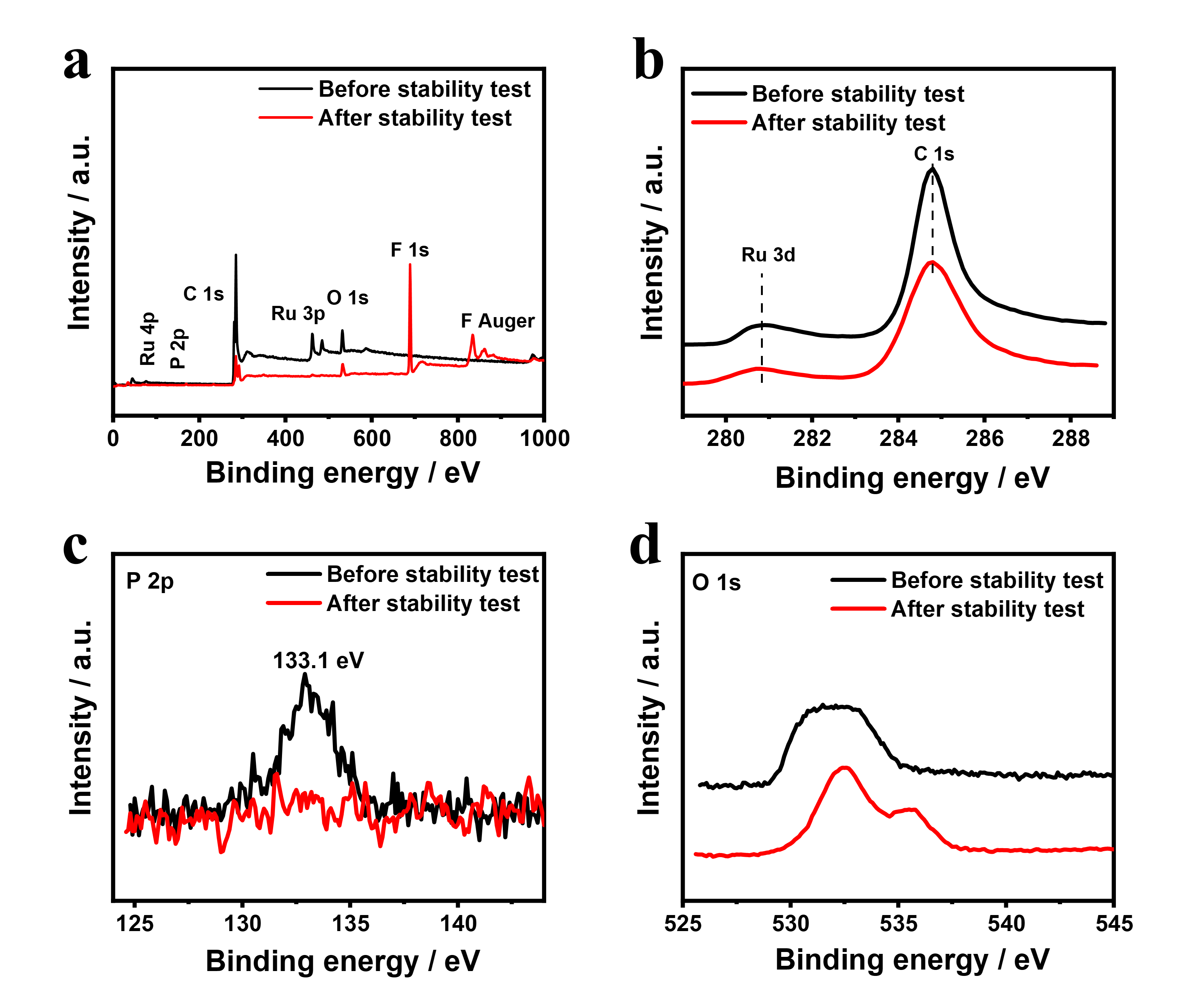


**Figure 19.** (a) Survey of XPS spectra for the as-prepared c/a-Ru/PC material before and after stability test. High-resolution XPS spectra of (b) Ru 3d and C 1s, (c) P 2p, and (d) O 1s for c/a-Ru/PC material before and after stability test.


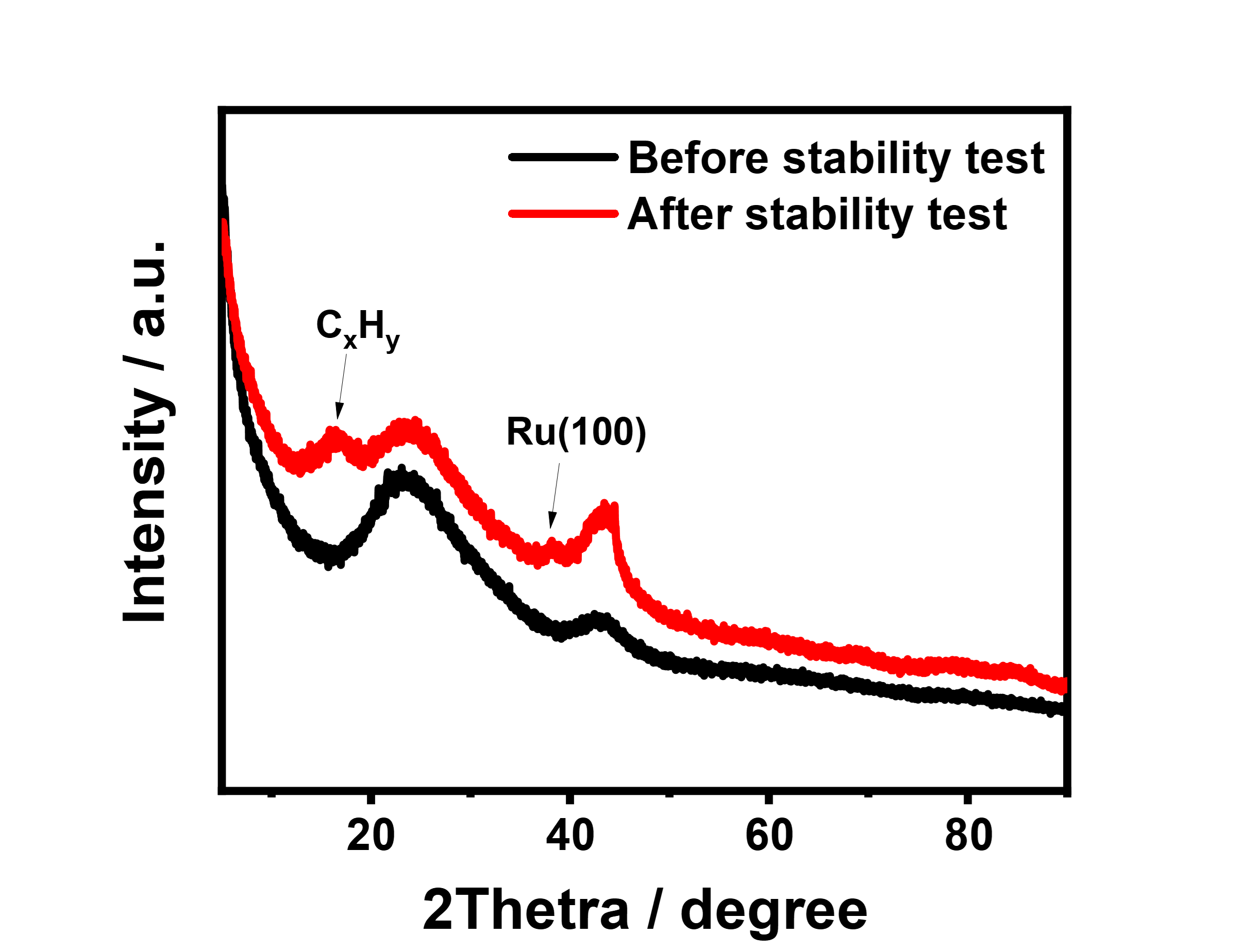


**Figure 20.** XRD patterns for the as-prepared c/a-Ru/PC material before and after stability test.


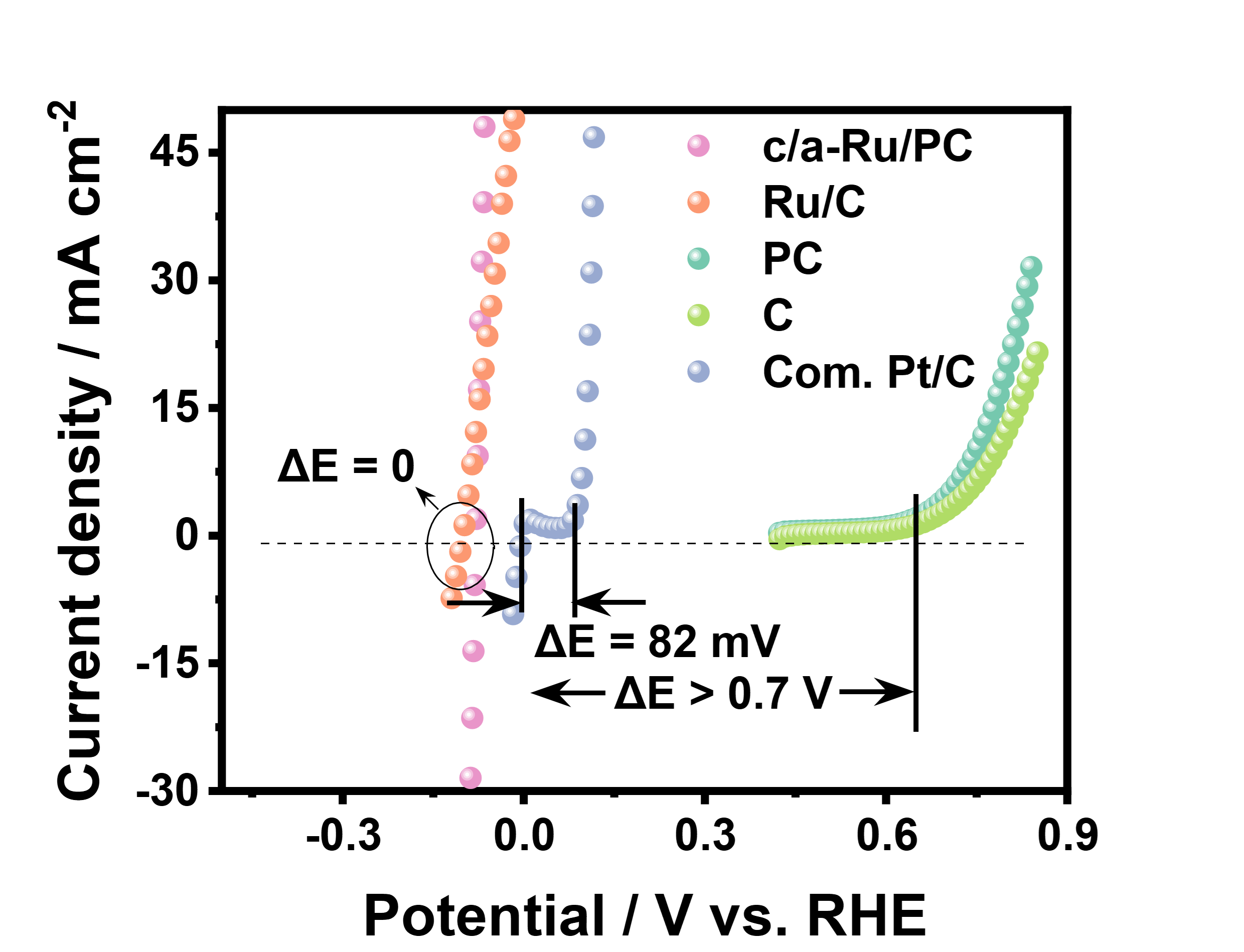


**Figure 21.** LSV curves for different catalysts in 1 M KOH + 1 M N_2_H_4_ solution, in which c/a-Ru/PC and Ru/C enable zero-drag hybrid seawater electrolysis from HER to HzOR regions.


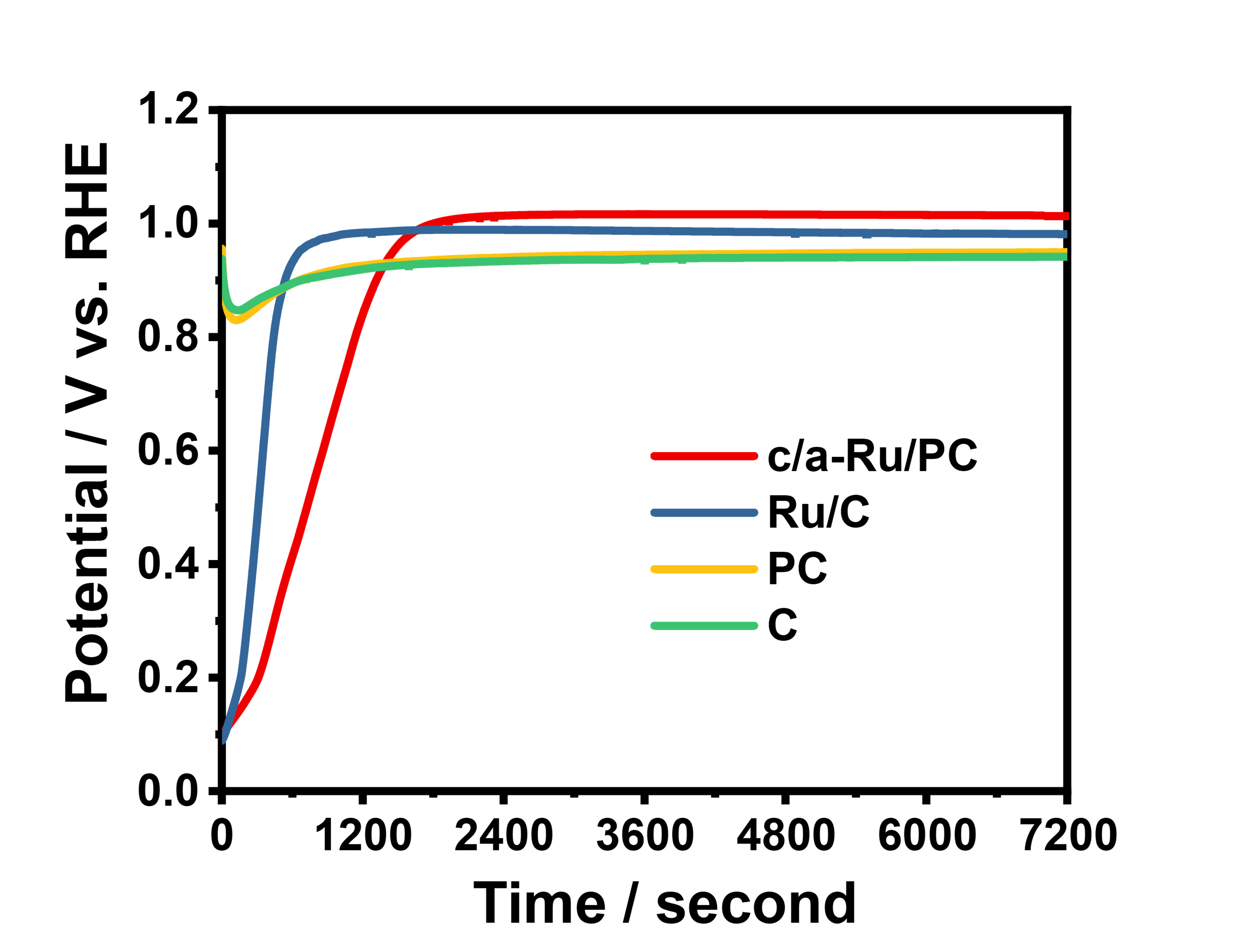


**Figure 22.** The open circuit potential measurements for 7200 s on c/a-Ru/PC, Ru/C, PC, and C catalysts in 1 M KOH electrolyte.


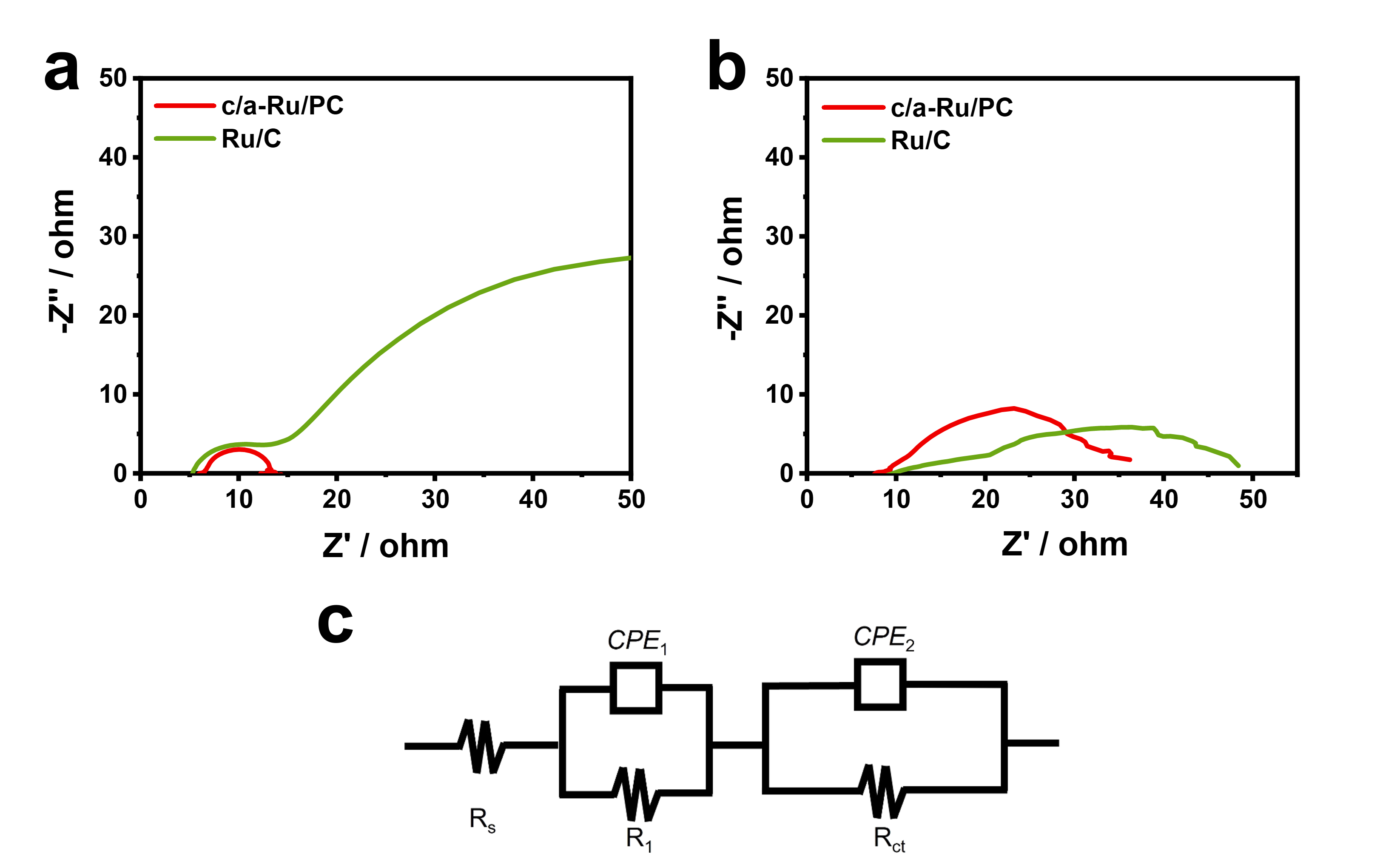


**Figure 23.** Nyquist plots of different catalyst electrodes obtained from electrochemical AC impedance measurement at the potential of (a) -0.12 V *vs.* RHE in 1M KOH, (b) -0.157 V *vs.* RHE in 1M KOH+1M N_2_H_4_ solution, and (c) the equivalent circuit diagram.


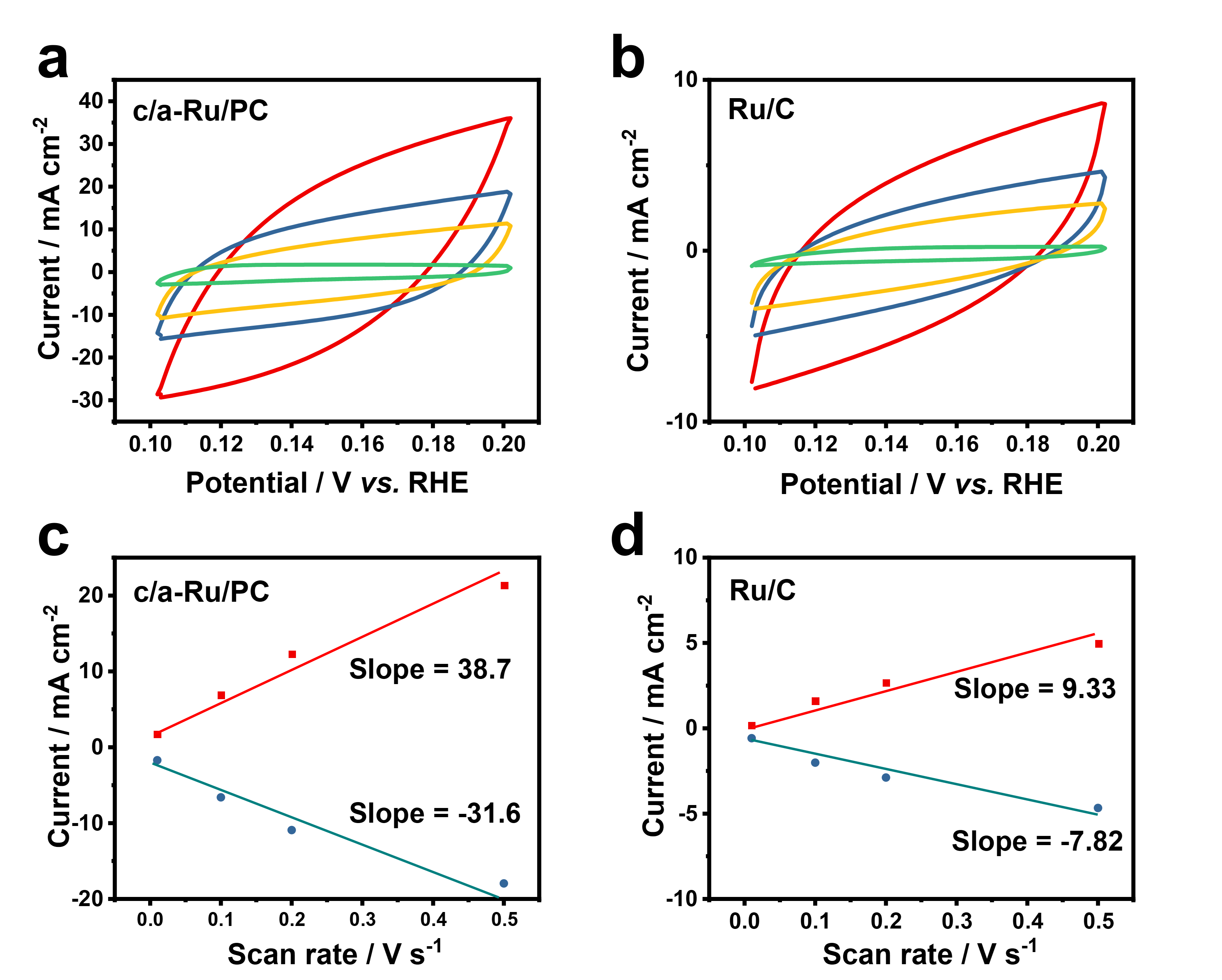


**Figure 24.** The capacitance currents as a function of the scan rates for the evaluation of electrical double-layer capacitor (C_dl_) and the electrochemical active surface area (ECSA) on (a and c) c/a-Ru/PC, (b and d)Ru/C electrocatalysts.


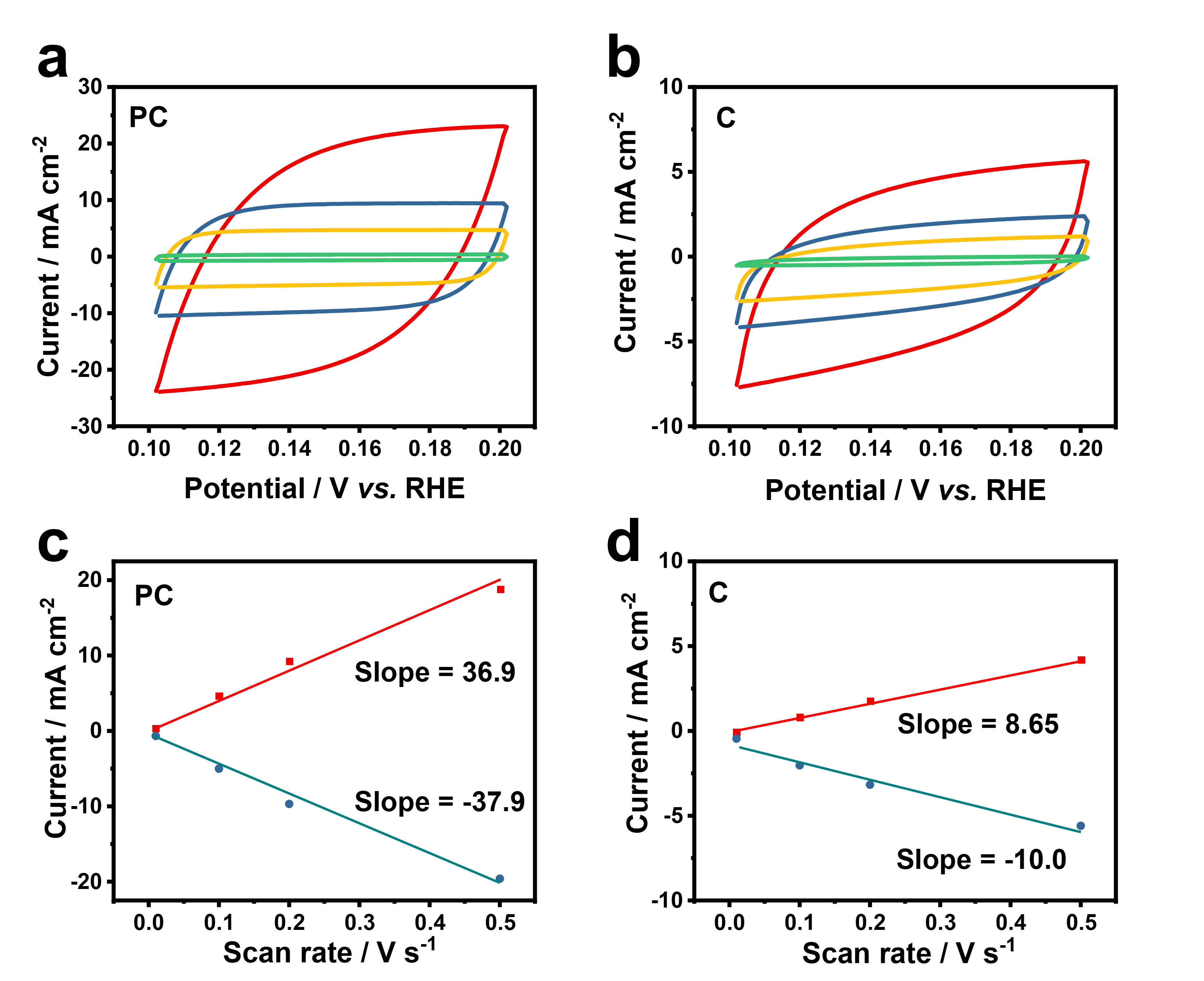


**Figure 25.** The capacitance currents as a function of the scan rates for the evaluation of electrical double-layer capacitor (C_dl_) and the electrochemical active surface area (ECSA) on (a and c) PC, (b and d) C electrocatalysts.

In this work, a non-Faraday potential region (0.1~0.2 V vs. RHE) was used to calculate C_dl_ and ECSA. The anodic/cathodic currents obtained at 0.15 V *vs.* RHE were plotted against the scan rates, following with the implement of a linear fitting to determine the slope. The mean of the absolute values of slopes was taken to calculate the C_dl_ by this formula: C_dl_ = (ja + |jc|)/(2v) = Δj/(2v), in which ja and jc represent the anodic and cathodic current, respectively, and v stands for the scanning rate. The ECSA was further calculated via this equation: ECSA = C_dl_/Cs, in which Cs represents the standard capacitance of a flat electrode (40 μF cm^-2^).


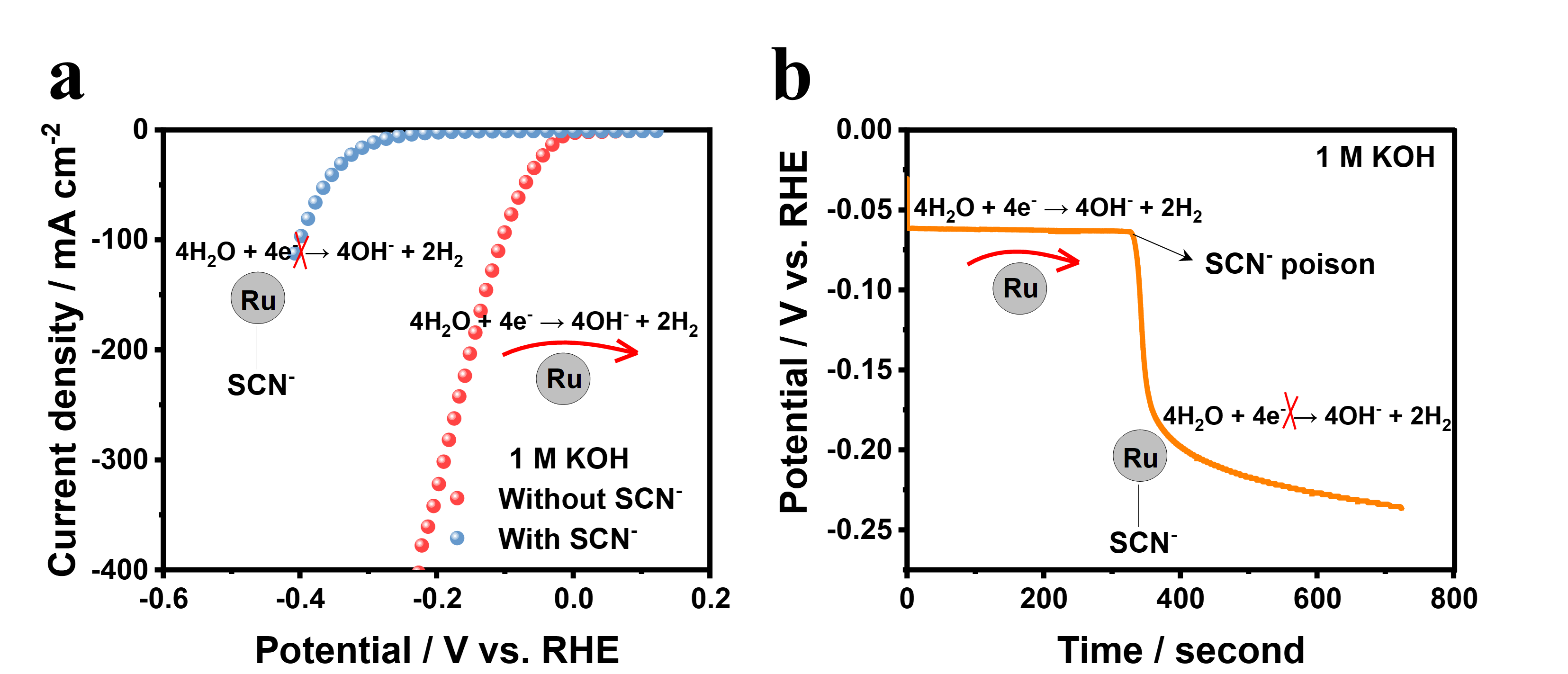


**Figure 26.** (a) LSV curves of c/a-Ru/PC before and after the addition of SCN^-^ ions in the 1 M KOH solution. (b) chronopotentiometry curve of c/a-Ru/PC before and after adding SCN^-^ ions to 1 M KOH solution. The insets in both figures illustrate the HER pathway with and without SCN^-^ ions.


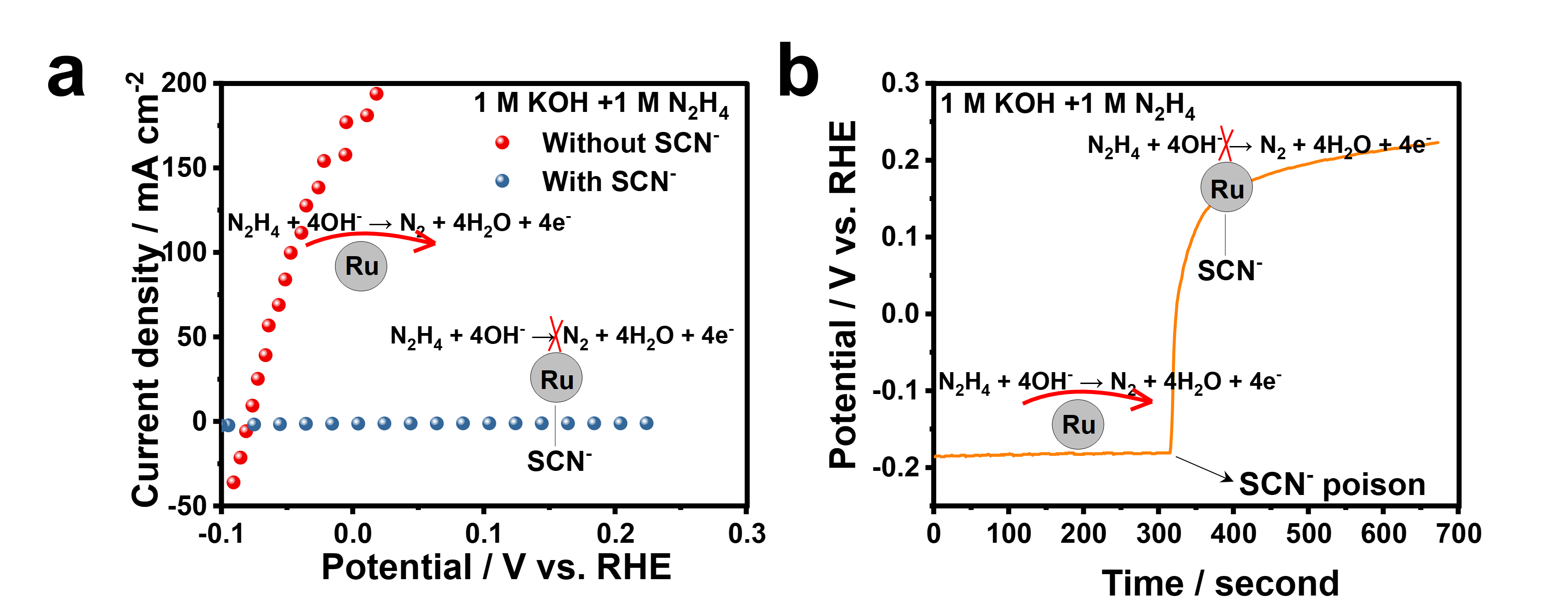


**Figure 27.** (a) LSV curves of c/a-Ru/PC before and after the addition of SCN^-^ ions in the 1 M KOH + 1 M N_2_H_4_ solution. (b) chronopotentiometry curve of c/a-Ru/PC before and after adding SCN^-^ ions to 1 M KOH + 1 M N_2_H_4_ solution. The insets in both figures illustrate the HzOR pathway with and without SCN^-^ ions.


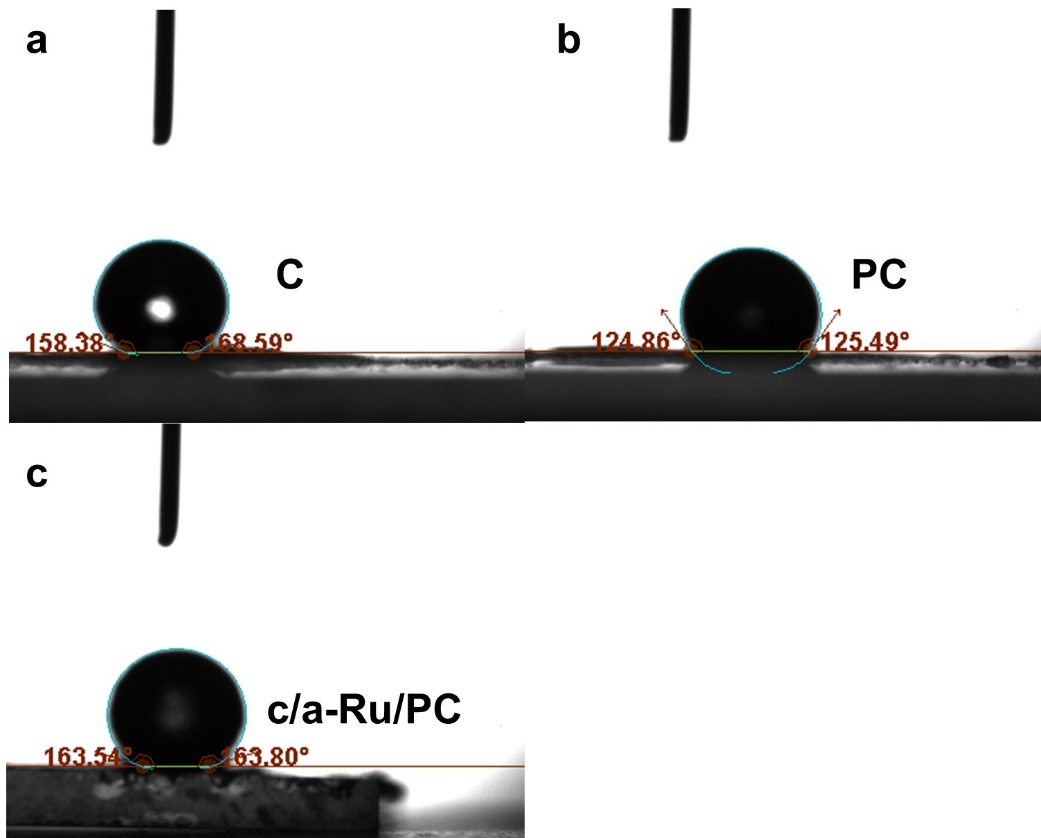


**Figure 28.** Contact angle test of (a) C, (b) PC, and (c) c/a-Ru/PC samples.


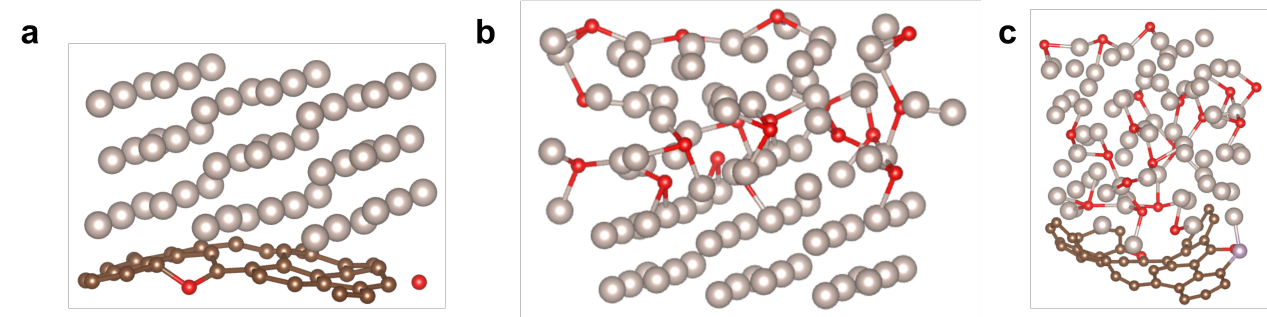


**Figure 29.** The optimized structural models of (a) crystalline state Ru-PC heterojunction, (b) amorphous-crystalline Ru heterojunction, and (c) amorphous Ru-PC heterojunction.

**
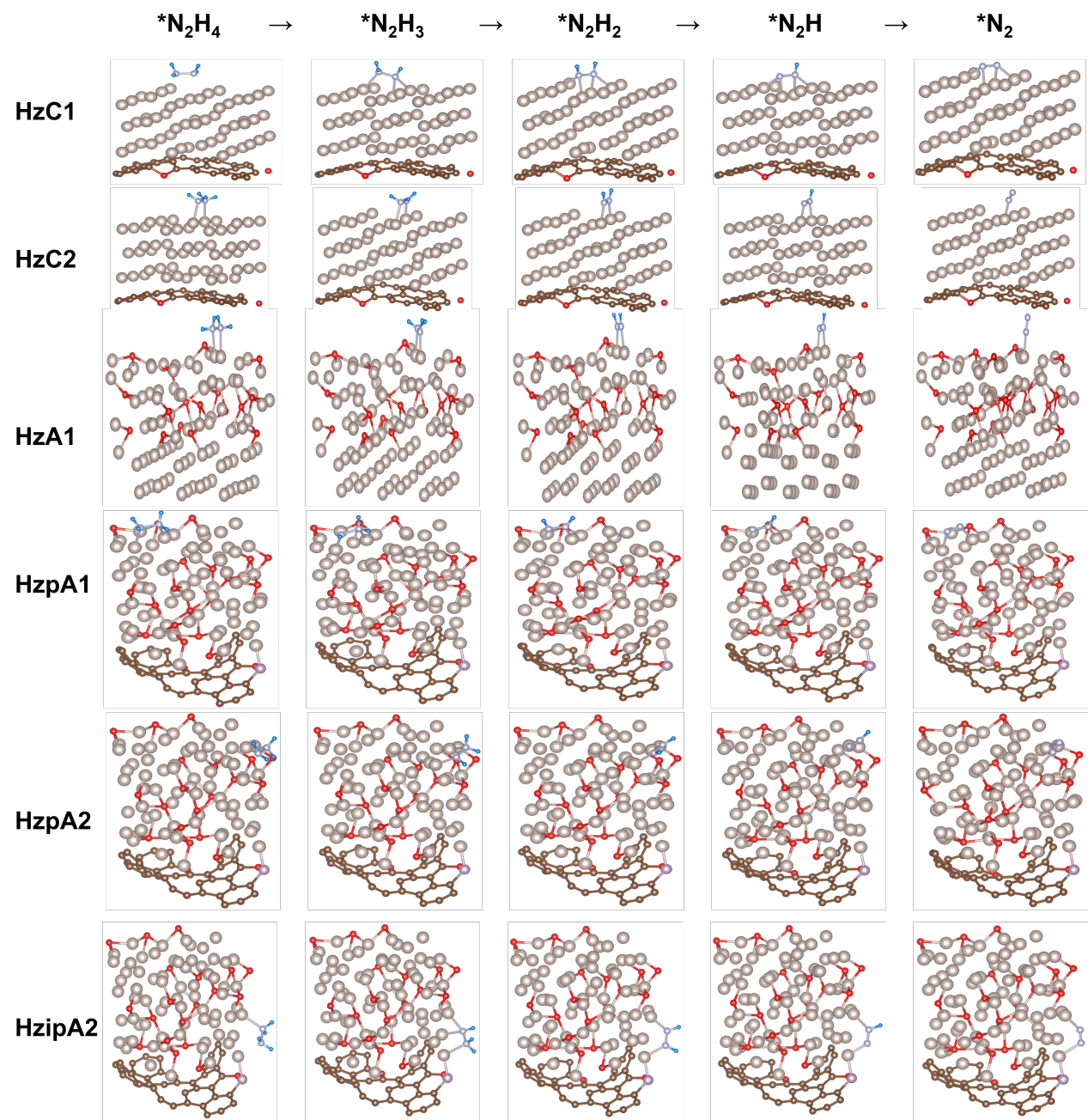
**

**Figure 30.** Adsorption of N_x_H_y_ species for HzOR on different Ru sites. Ru, C, O, N, P, and H elements are marked by gray, brown, red, cambridge blue, purple, and blue respectively.


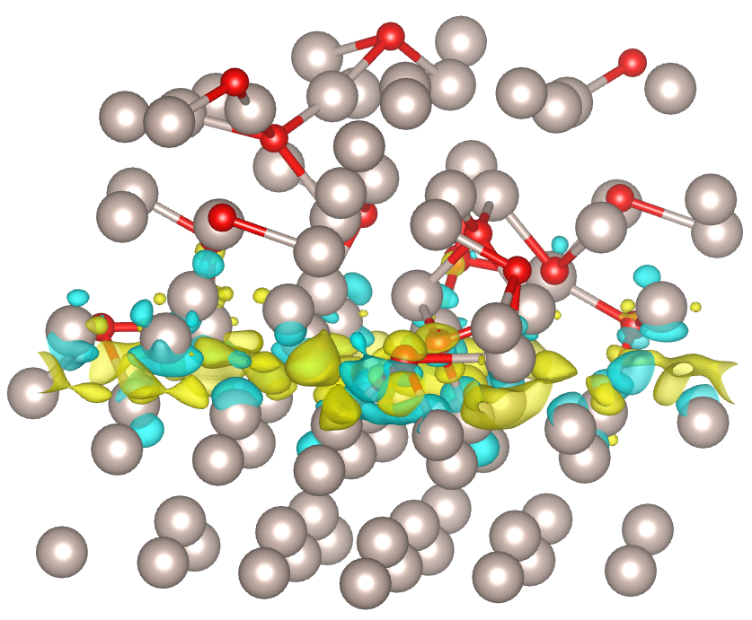


**Figure 31.** Charge density difference of c/a-Ru/PC with the isosurface level of 0.01 e Å^−3^.


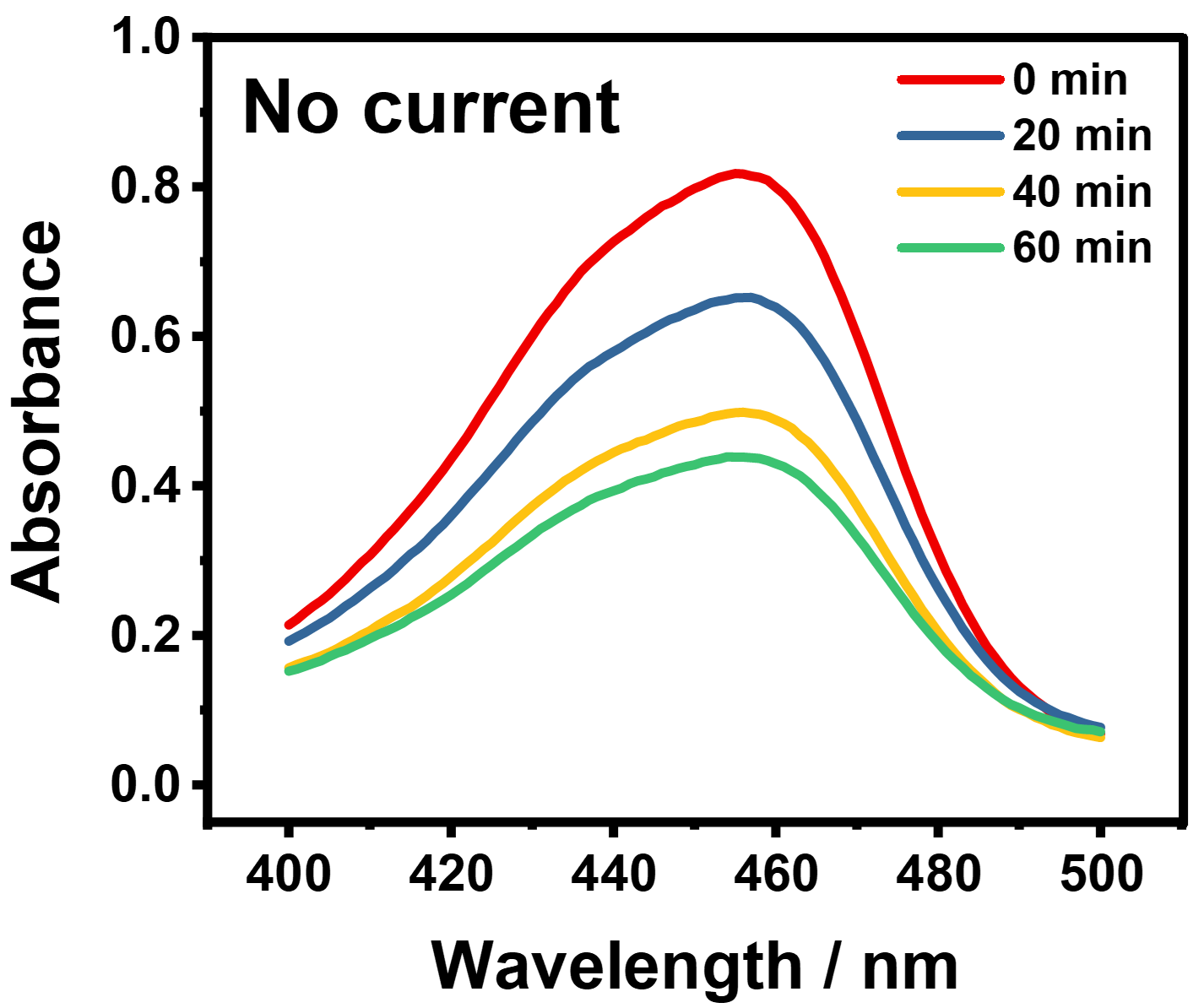


**Figure 32.** Uv-visible (UV) absorption spectra of N_2_H_4_ sewage degradation along with time without current applied.


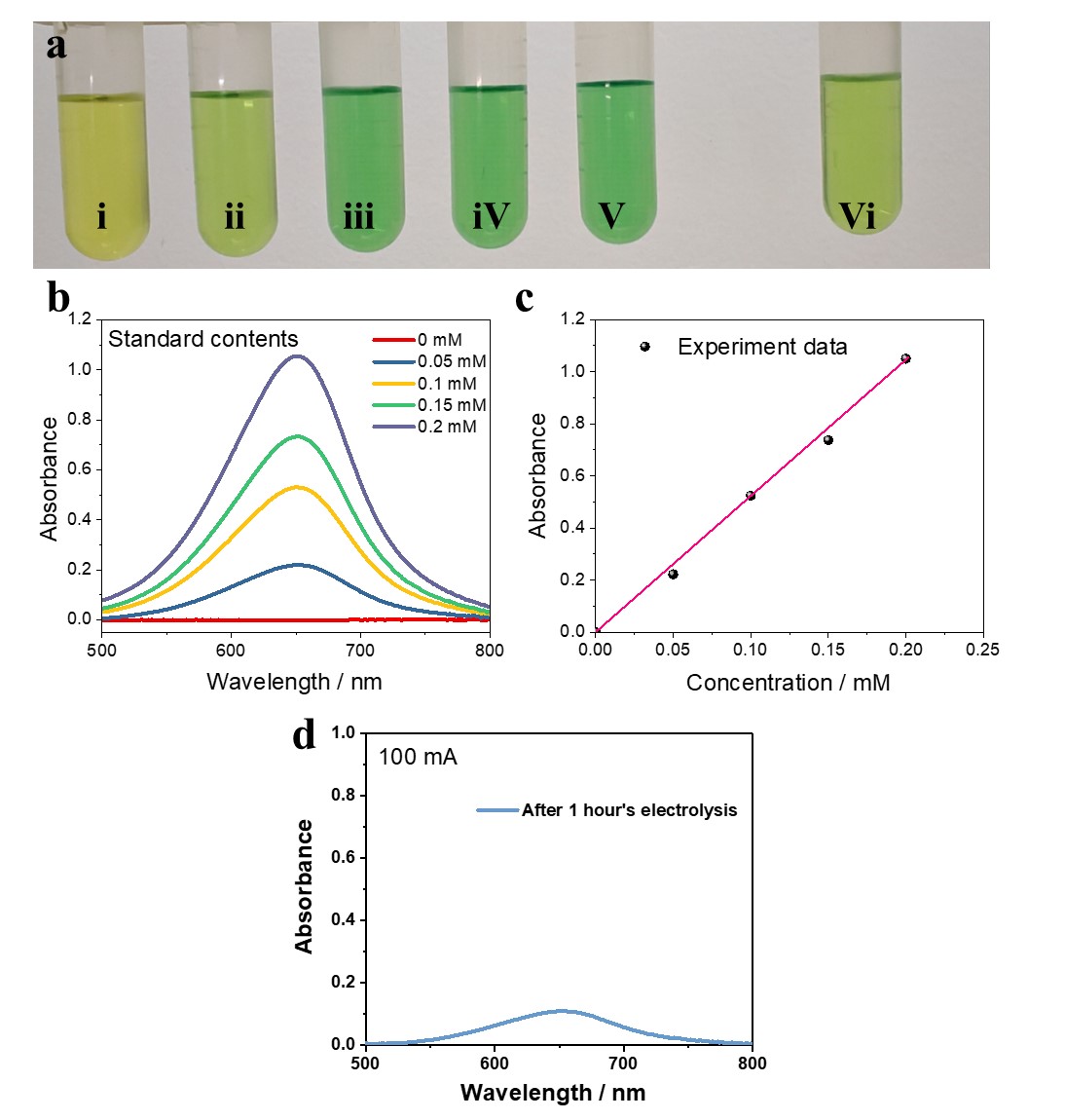


**Figure 33.** ‌(a)‌ Images of standard ammonium solutions at different concentrations (i-v) and the electrolyzed test solution after 1-hour electrolysis at 100 mA (vi). ‌(b)‌ UV absorption spectra of the corresponding standard ammonium solutions. ‌(c)‌ UV absorption spectrum of the electrolyzed test solution. ‌(d)‌ Absorbance-concentration calibration curve for ammonium standards.

**Table 1.** EXAFS fitting parameters at the Ru K-edge of various samples (S_0_^2^=0.86). *^a^*C. N.: coordination numbers; *^b^R*: bond distance; *^c^σ*^2^: Debye-Waller factors; *^d^*Δ*E*_0_: the inner potential correction. *^e^R* factor: goodness of fit. *The experimental EXAFS fit by fixing C. N. as the known crystallographic value.

| samples | path | C. N.^[a]^ | R (Å) ^[b]^ | σ^2^ (× 10^−3^ Å^2^) ^[c]^ | ΔE (eV) ^[d]^ | R factor^[e]^ |
| --- | --- | --- | --- | --- | --- | --- |
| Ru foil | Ru-Ru | 12* | 2.67±0.01 | 4.0±0.7 | -5.6±1.2 | 0.01 |
| RuO_2_ | Ru-O | 6.0±0.9 | 1.99±0.02 | 5.6±4.1 | -1.7±4.0 | 0.01 |
|  | Ru-O-Ru | 1.7±0.3 | 3.14±0.02 | 2.8±2.3 |  |  |
|  | Ru-O-O | 2.0±0.3 | 3.45±0.02 | 8.6±2.3 |  |  |
| c/a-Ru/PC | Ru-O | 3.3±0.7 | 1.97±0.02 | 6.0±5.1 | 0.4±1.7 | 0.02 |
|  | Ru-P | 1.2±0.2 | 2.21±0.02 | 7.2±1.6 |  |  |
|  | Ru-Ru | 4.9±1.2 | 2.68±0.03 | 4.5±1.9 |  |  |

**Table 2.** Comparison of the HER performance on different Ru-based electrocatalysts.

| Electrocatalyst | Catalyst loading (mg_Ru_ cm^-2^) | Electrolyte | Overpotential@10 mA cm^-2^ (mV) | Tafel slope mV dec^-1^ | Reference |
| --- | --- | --- | --- | --- | --- |
| c/a-Ru/PC | 0.03 | 1 M KOH | 24.7 | 41.3 | This work |
| Ru@V-RuO_2_/C HMS | 0.28 | 1 M KOH | 47 | 44.8 | ^[6]^ |
| Ru_2_ SACs@FeCo-LDH | - | 1 M KOH | 84 mV @500 mA cm^-2^ | 53 | ^[7]^ |
| RuTe_2_ | - | 1 M KOH | 34 | 28 | ^[8]^ |
| CC@WS_2_/Ru-450 | 0.003 | 1 M KOH | 32.1 | 53.2 | ^[9]^ |
| MoP-Ru_2_P/NPC | - | 1 M KOH | 47 | 36.9 | ^[10]^ |
| RuNP-RuSA@CFN-800 | 0.16 | 1 M KOH | 33 | 37.2 | ^[11]^ |
| Ru-RuO_2_@NPC | 0.058 | 0.5 M H_2_SO_4_ | 68 | 56 | ^[12]^ |
| (Ru–Co)O_x_ | 0.227 | 1 M KOH | 44.1 | 23.5 | ^[13]^ |
| Ru-G/CC | 0.57 | 1 M KOH | 40 | 76 | ^[14]^ |
| Ru-NiFe-P | - | 1 M KOH | 44 | 80 | ^[15]^ |
| Ru-WO_3-x_ /CP | - | 1.0M PBS | 19 | 41 | ^[16]^ |
| Ru-NiCoP/NF | - | 1 M KOH | 44 | 45.4 | ^[17]^ |
| RuP_2_@NPC | 0.233 | 1 M KOH | 52 | 38 | ^[18]^ |
| Ru/Cu_2_O/CF | - | 1 M KOH | 31 | 50 | ^[19]^ |
| RuP/CoNiP_4_O_12_/ CC | - | 1 M KOH | 27 | 28.5 | ^[20]^ |
| Ru-(Ni/Fe)C_2_O_4_ | - | 1 M KOH | 42 | 39 | ^[21]^ |
| Ru-FeP_4_/IF |  | 1 M KOH | 110 mV @100 mA cm^-2^ | 51.6 | ^[22]^ |
| Ru–MnFeP/NF | - | 1 M KOH | 35 | 36 | ^[23]^ |

**Table 3.** Comparison of the HzOR performance on different Ru-based

| Electrocatalyst | Catalyst loading (mg_Ru_ cm^-2^) | Electrolyte | Potential [V_RHE_] | Reference |
| --- | --- | --- | --- | --- |
| c/a-Ru/PC | 0.03 | 1 M KOH + 1 M N_2_H_4_ | -0.076 @10 mA cm^-2^ | This work |
| Ru/Co_3_O_4_ | 0.031 | 1 M KOH + 1 M N_2_H_4_ | -0.024 @100 mA cm^-2^ | ^[24]^ |
| Ru1-NiCoP | 0.00457 | 1 M KOH + 0.3 M N_2_H_4_ | -0.06 @10 mA cm^-2^ | ^[25]^ |
| CC@WS_2_/Ru-450 | 0.003 | 1 M KOH + 1 M N_2_H_4_ | -0.074 @10 mA cm^-2^ | ^[9]^ |
| Ru/MPNC | 0.2 | 1 M KOH + 0.5 M N_2_H_4_ | -0.039 @10 mA cm^-2^ | ^[26]^ |
| Ru-(Ni/Fe)C_2_O_4_ | - | 1 M KOH + 0.1 M N_2_H_4_ | -0.096 @10 mA cm^-2^ | ^[21]^ |
| Ru-FeP_4_/IF | - | 1 M KOH + 0.5 M N_2_H_4_ | 0.027 @100 mA cm^-2^ | ^[22]^ |

electrocatalysts.

**Table 4.** Fitting data of equivalent circuit in the electrochemical impedance spectroscopy (EIS) for different catalysts at the potential of -0.12 V *vs.* RHE in 1 M KOH solution.

|  | c/a-Ru/PC | Ru/C |
| --- | --- | --- |
| R_s_ / ohm | 6.0 | 5.3 |
| CPE_1_ / S sec^0.7^ | 0.0088 | 0.00011 |
| R_1_ | 6.76 | 9.17 |
| CPE_2_ / S sec^0.7^ | 0.0050 | 0.0021 |
| R_ct_ / ohm | 6.63 | 75.2 |

**Notes:** R_ct_, R_s_, R_1_, and W in this table represent the resistance caused by charge transfer, electrolyte resistance, resistance from electrode porosity for ions transfer, and Warburg resistance, respectively. CPE is a constant phase angle element.

**Table 5.** Fitting data of equivalent circuit in the electrochemical impedance spectroscopy (EIS) for different catalysts at the potential of -0.157 V *vs.* RHE in 1 M KOH + 1 M N_2_H_4_ solution.

|  | c/a-Ru/PC | Ru/C |
| --- | --- | --- |
| R_s_ / ohm | 7.7 | 7.8 |
| CPE_1_ / S sec^0.7^ | 0.00031 | 8.6*10^-5^ |
| R_1_ | 1.483 | 6.1 |
| CPE_2_ / S sec^0.7^ | 0.0037 | 0.0018 |
| R_ct_ / ohm | 25.5 | 29.5 |

**Notes:** R_ct_, R_s_, R_1_, and W in this table represent the resistance caused by charge transfer, electrolyte resistance, resistance from electrode porosity for ions transfer, and Warburg resistance, respectively. CPE is a constant phase angle element.

**Reference**

[1] G. Kresse, D. Joubert, From ultrasoft pseudopotentials to the projector augmented-wave method, *Physical Review B* **1999**, *59*, 1758-1775. <https://doi.org/10.1103/PhysRevB.59.1758>.

[2] P. Giannozzi, S. Baroni, N. Bonini, M. Calandra, R. Car, C. Cavazzoni, D. Ceresoli, G. L. Chiarotti, M. Cococcioni, I. Dabo, A. D. Corso, S. Fabris, G. Fratesi, S. d. Gironcoli, R. Gebauer, U. Gerstmann, C. Gougoussis, A. Kokalj, M. Lazzeri, L. Martin-Samos, N. Marzari, F. Mauri, R. Mazzarello, S. Paolini, A. Pasquarello, L. Paulatto, C. Sbraccia, S. Scandolo, G. Sclauzero, A. P. Seitsonen, A. Smogunov, P. Umari, R. M. Wentzcovitch, Quantum ESPRESSO: a modular and open-source software project for quantum simulations of materials, *Journal of Physics: Condensed Matter* **2009**, *21*, 395502. <https://doi.org/10.1088/0953-8984/21/39/395502>.

[3] J. P. Perdew, K. Burke, M. Ernzerhof, Generalized gradient approximation made simple, *Physics letter journal* **1997**, *77*, 3865–3868. <https://doi.org/10.1103/PhysRevLett.77.3865>.

[4] H. J. Monkhorst, J. D. Pack, Special points for Brillouin-zone integrations, *Physical Review B* **1976**, *13*, 5188-5192. <https://doi.org/10.1103/PhysRevB.13.5188>.

[5] J. K. Nørskov, J. Rossmeisl, A. Logadottir, L. Lindqvist, J. R. Kitchin, T. Bligaard, H. Jónsson, Origin of the overpotential for oxygen reduction at a fuel-cell cathode, *The Journal of Physical Chemistry B* **2004**, *108*, 17886–17892. <https://doi.org/10.1021/jp047349j>.

[6] Y. Li, W. Wang, M. Cheng, Y. Feng, X. Han, Q. Qian, Y. Zhu, G. Zhang, Arming Ru with oxygen-vacancy-enriched RuO_2_ sub-nanometer skin activates superior bifunctionality for pH-universal overall water splitting, *Advanced Materials* **2023**, *35*, 2206351. <https://doi.org/10.1002/adma.202206351>.

[7] X. Mu, X. Gu, S. Dai, J. Chen, Y. Cui, Q. Chen, M. Yu, C. Chen, S. Liu, S. Mu, Breaking the symmetry of single-atom catalysts enables an extremely low energy barrier and high stability for large-current-density water splitting, *Energy & Environmental Science* **2022**, *15*, 4048-4057. <https://doi.org/10.1039/d2ee01337a>.

[8] B. Tang, X. Yang, Z. Kang, L. Feng, Crystallized RuTe_2_ as unexpected bifunctional catalyst for overall water splitting, *Applied Catalysis B-Environmental* **2020**, *278*, 119281. <https://doi.org/10.1016/j.apcatb.2020.119281>.

[9] J. Li, Y. Li, J. Wang, C. Zhang, H. Ma, C. Zhu, D. Fan, Z. Guo, M. Xu, Y. Wang, H. Ma, Elucidating the critical role of ruthenium single atom sites in water dissociation and dehydrogenation behaviors for robust hydrazine oxidation-boosted alkaline hydrogen evolution, *Advanced Functional Materials* **2022**, *32*, 2109439. <https://doi.org/10.1002/adfm.202109439>.

[10] Y. Gao, Z. Chen, Y. Zhao, W. Yu, X. Jiang, M. He, Z. Li, T. Ma, Z. Wu, L. Wang, Facile synthesis of MoP-Ru_2_P on porous N, P co-doped carbon for efficiently electrocatalytic hydrogen evolution reaction in full pH range, *Applied Catalysis B-Environmental* **2022**, *303*, 120879. <https://doi.org/10.1016/j.apcatb.2021.120879>.

[11] T. Luo, J. Huang, Y. Hu, C. Yuan, J. Chen, L. Cao, K. Kajiyoshi, Y. Liu, Y. Zhao, Z. Li, Y. Feng, Fullerene lattice-confined Ru nanoparticles and single atoms synergistically boost electrocatalytic hydrogen evolution reaction, *Advanced Functional Materials* **2023**, *33*, 2213058. <https://doi.org/10.1002/adfm.202213058>.

[12] N. Wang, S. Ning, X. Yu, D. Chen, Z. Li, J. Xu, H. Meng, D. Zhao, L. Li, Q. Liu, B. Lu, S. Chen, Graphene composites with Ru-RuO_2_ heterostructures: highly efficient Mott-Schottky-type electrocatalysts for pH-universal water splitting and flexible zinc-air batteries, *Applied Catalysis B-Environmental* **2022**, *302*, 120838. <https://doi.org/10.1016/j.apcatb.2021.120838>.

[13] C. Wang, L. Qi, Heterostructured inter-doped ruthenium-cobalt oxide hollow nanosheet arrays for highly efficient overall water splitting, *Angewandte Chemie-International Edition* **2020**, *59*, 17219-17224. <https://doi.org/10.1002/anie.202005436>.

[14] M. You, X. Du, X. Hou, Z. Wang, Y. Zhou, H. Ji, L. Zhang, Z. Zhang, S. Yi, D. Chen, In-situ growth of ruthenium-based nanostructure on carbon cloth for superior electrocatalytic activity towards HER and OER, *Applied Catalysis B-Environmental* **2022**, *317*, 121729. <https://doi.org/10.1016/j.apcatb.2022.121729>.

[15] M. Qu, Y. Jiang, M. Yang, S. Liu, Q. Guo, W. Shen, M. Li, R. He, Regulating electron density of NiFe-P nanosheets electrocatalysts by a trifle of Ru for high-efficient overall water splitting, *Applied Catalysis B-Environmental* **2020**, *263*, 118324. <https://doi.org/10.1016/j.apcatb.2019.118324>.

[16] J. Chen, C. Chen, M. Qin, B. Li, B. Lin, Q. Mao, H. Yang, B. Liu, Y. Wang, Reversible hydrogen spillover in Ru-WO_3-x_ enhances hydrogen evolution activity in neutral pH water splitting, *Nature Communications* **2022**, *13*, 5382. <https://doi.org/10.1038/s41467-022-33007-3>.

[17] D. Chen, R. Lu, Z. Pu, J. Zhu, H.-W. Li, F. Liu, S. Hu, X. Luo, J. Wu, Y. Zhao, S. Mu, Ru-doped 3D flower-like bimetallic phosphide with a climbing effect on overall water splitting, *Applied Catalysis B-Environmental* **2020**, *279*, 119396. <https://doi.org/10.1016/j.apcatb.2020.119396>.

[18] Z. Pu, I. S. Amiinu, Z. Kou, W. Li, S. Mu, RuP_2_‐Based Catalysts with Platinum‐like Activity and Higher Durability for the Hydrogen Evolution Reaction at All pH Values, *Angewandte Chemie International Edition* **2017**, *56*, 11559-11564. <https://doi.org/10.1002/anie.201704911>.

[19] P. Shen, B. Zhou, Z. Chen, W. Xiao, Y. Fu, J. Wan, Z. Wu, L. Wang, Ruthenium-doped 3D Cu_2_O nanochains as efficient electrocatalyst towards hydrogen evolution and hydrazine oxidation, *Applied Catalysis B: Environmental* **2023**, *325*, 122305. <https://doi.org/10.1016/j.apcatb.2022.122305>.

[20] J. Zhao, Y. Zhang, Y. Xia, B. Zhang, Y. Du, B. Song, H. Wang, S. Li, P. Xu, Strong phosphide-metaphosphate interaction in RuP/CoNiP_4_O_12_ for enhanced electrocatalytic water splitting, *Applied Catalysis B-Environment and Energy* **2023**, *328*, 122447. <https://doi.org/10.1016/j.apcatb.2023.122447>.

[21] J. Zhao, H. Guo, Q. Zhang, Y. Li, L. Gu, R. Song, Trace Ru atoms implanted into a Ni/Fe-based oxalate solid-solution-like with high-indexed facets for energy-saving overall seawater electrolysis assisted by hydrazine, *Applied Catalysis B: Environmental* **2023**, *325*, 122354. <https://doi.org/10.1016/j.apcatb.2022.122354>.

[22] T. Cui, J. Chi, J. Zhu, X. Sun, J. Lai, Z. Li, L. Wang, Tuning the size and chemisorption of FeP_4_ by trace Ru doping for hydrazine-assisted hydrogen evolution in seawater at large-current-density, *Applied Catalysis B-Environmental* **2022**, *319*, 121950. <https://doi.org/10.1016/j.apcatb.2022.121950>.

[23] D. Chen, Z. Pu, R. Lu, P. Ji, P. Wang, J. Zhu, C. Lin, H.-W. Li, X. Zhou, Z. Hu, F. Xia, J. Wu, S. Mu, Ultralow Ru loading transition metal phosphides as high-efficient bifunctional electrocatalyst for a solar-to-hydrogen generation system, *Advanced Energy Materials* **2020**, *10*, 2000814. <https://doi.org/10.1002/aenm.202000814>.

[24] Y. Zhai, C. Jin, Q. Xia, W. Han, J. Wu, X. Zhao, X. Zhang, Atomically confined Ru sites in octahedral Co_3_O_4_ for high-efficiency hydrazine oxidation, *Advanced Functional Materials* **2023**, DOI: 10.1002/adfm.202311063, 2311063. <https://doi.org/10.1002/adfm.202311063>.

[25] Y. Hu, T. Chao, Y. Li, P. Liu, T. Zhao, G. Yu, C. Chen, X. Liang, H. Jin, S. Niu, W. Chen, D. Wang, Y. Li, Cooperative Ni(Co)-Ru-P sites activate dehydrogenation for hydrazine oxidation assisting self-powered H_2_ production, *Angewandte Chemie-International Edition* **2023**, *62*, e202308800. <https://doi.org/10.1002/anie.202308800>.

[26] J. Wang, X. Guan, H. Li, S. Zeng, R. Li, Q. Yao, H. Chen, Y. Zheng, K. Qu, Robust Ru-N metal-support interaction to promote self-powered H_2_ production assisted by hydrazine oxidation, *Nano Energy* **2022**, *100*, 107467. <https://doi.org/10.1016/j.nanoen.2022.107467>.
